# OSA Initiates Histone Lactylation That Drives PDE4B/FUS/AGT Axis to Pulmonary Hypertension

**DOI:** 10.1111/cpr.70145

**Published:** 2025-11-17

**Authors:** Li Yang, Qing Ni, Yan He, Shijie Liu, Lulu Gan, Anni Dai, Yang Hu, Qian Liu, Xueling Yang, Jiqian Li, Yi Tao, Yunyu Li, Mingyue Xu

**Affiliations:** ^1^ The Affiliated Yan’an Hospital of Kunming Medical University Kunming Hypertension Center Kunming City China; ^2^ Kunming Technical Diagnosis and Treatment Center for Refractory Hypertension Kunming City China

**Keywords:** histone lactylation, OSA‐related hypertension, oxidative stress, PASMC remodelling, PDE4B

## Abstract

Obstructive sleep apnea (OSA) is strongly associated with an increased risk of hypertension; however, the molecular mechanisms linking these two conditions remain incompletely understood. In this study, we identified phosphodiesterase 4B (PDE4B) as a key mediator in the development of OSA‐related hypertension. Using integrated bioinformatics analysis and experimental validation, we found that PDE4B expression was significantly elevated in both cell and animal models of OSA combined with pulmonary hypertension. Functional studies demonstrated that PDE4B promotes pulmonary artery smooth muscle cell (PASMC) proliferation and migration, contributing to vascular remodelling. Mechanistically, we uncovered that lactate accumulation under hypoxic conditions induces histone lactylation at the PDE4B promoter, enhancing its transcriptional activity. Furthermore, PDE4B was shown to regulate the phosphorylation and nuclear translocation of FUS, which binds to the angiotensinogen (AGT) promoter and enhances AGT expression, thereby promoting pulmonary hypertension. These findings reveal a novel PDE4B‐FUS‐AGT signalling axis driven by epigenetic modifications in OSA‐induced hypertension, offering potential therapeutic targets for patients with this comorbidity.

## Graphical abstract

This study illustrates how chronic intermittent hypoxia (CIH) in obstructive sleep apnea (OSA) leads to hypertension via increased oxidative stress and mitochondrial dysfunction in pulmonary artery smooth muscle cells (PASMCs), causing glycolytic dysregulation. Lactate accumulation enhances histone lactylation, upregulating phosphodiesterase 4B (PDE4B), which modulates nucleocytoplasmic trafficking through FUS phosphorylation, promoting angiotensinogen (AGT) biosynthesis and PASMC remodelling. Therapeutic interventions, including PDE4B silencing and antioxidant treatment, mitigate these effects, normalising glycolysis and inhibiting the PDE4B/FUS/AGT axis. The graphic highlights the pathway from CIH exposure to potential treatments, guiding viewers through the mechanisms and therapeutic strategies.

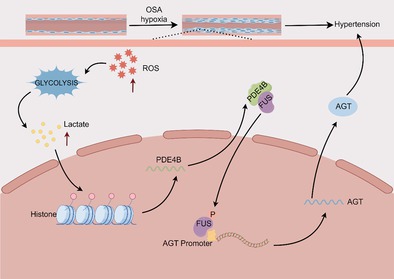

## 1 Introduction

Obstructive sleep apnea (OSA) is a common sleep disorder characterised by repeated obstruction of the upper airway during sleep, leading to inadequate ventilation and subsequently causing intermittent hypoxia, hypercapnia, and disrupted sleep architecture [1]. This disease not only severely impacts patients' sleep quality but is also closely related to the occurrence and development of various cardiovascular diseases, with pulmonary hypertension being one of its most common comorbidities [2, 3]. Studies have shown that the prevalence of combined hypertension in OSA patients ranges from 50% to 80%, and in patients with resistant hypertension, the prevalence of OSA can be as high as 70%–85% [4]. Therefore, exploring the mechanisms underlying the interaction between OSA and pulmonary hypertension is of significant importance for reducing the risk of cardiovascular disease and improving patient outcomes.

Research has found that OSA combined with hypertension is closely related to the activation of the sympathetic nervous system, oxidative stress, and systemic inflammation caused by recurrent hypoxia [5, 6, 7]. Among these, oxidative stress has become one of the hot topics in cardiovascular disease research in recent years. Oxidative stress refers to an imbalance between oxidation and antioxidant systems in the body, leading to the excessive production of highly reactive molecules such as reactive oxygen species (ROS) and reactive nitrogen species (RNS). These not only directly damage biological macromolecules such as DNA, proteins, and lipids but also affect cellular metabolic pathways, including glycolysis [8]. Abnormal glycolysis further regulates the epigenetic of cells through the accumulation of metabolites such as lactate. Histone lactylation, a histone post‐translational modification discovered in recent years, provides new insights for understanding epigenetic regulation [9, 10]. Lactate molecules covalently bind to specific lysine residues on histones, forming lactylation modifications, which can influence the structure and function of chromatin, thereby regulating gene expression and potentially affecting cell fate. Currently, studies have found that histone lactylation modifications play a role in the development of hypoxic pulmonary hypertension [11, 12].

Phosphodiesterase 4B (PDE4B), an important signal transduction enzyme, is involved in regulating intracellular cyclic adenosine monophosphate (cAMP) levels, thereby influencing various physiological and pathological processes. In recent years, its role in inflammatory responses and airway smooth muscle function has gradually garnered attention [13, 14]. By degrading cAMP, PDE4B modulates the release of inflammatory mediators and smooth muscle contraction, potentially playing a significant role in the mechanism of hypertension induced by obstructive sleep apnea hypopnea syndrome (OSAHS). Furthermore, studies have shown that PDE4B promotes endothelial‐mesenchymal transition (EMT) through the regulation of the cAMP/PKA/CREB/BMPRII signalling pathway, thereby exacerbating pulmonary vascular remodelling and the development of pulmonary arterial hypertension [15]. Notably, there may be a certain relationship between PDE4B, oxidative stress and histone lactylation in OSA comorbid with hypertension. On one hand, PDE4B influences the cellular redox state by regulating the cAMP signalling pathway; on the other hand, histone lactylation modification under oxidative stress conditions may also affect the activity and expression of PDE4B. However, whether there is any interaction between them and how they influence OSA combined with hypertension and the potential mechanisms remain unclear.

Here, we investigated the mechanism of action of PDE4B in OSA combined with hypertension. We found that in OSA combined with hypertension, pulmonary artery smooth muscle cells (PASMCs) undergo oxidative stress and mitochondrial damage, leading to abnormal glycolysis, which further induces histone lactylation, thereby affecting the transcription and expression of PDE4B. Moreover, the knockout of PDE4B improved the phenotype of OSA combined with hypertension. Mechanistically, PDE4B promotes the transcription of AGT by enhancing the nuclear expression of FUS through its phosphorylation. In the future, with further elucidation of the functions and mechanisms of PDE4B, we have reason to believe that inhibitors targeting PDE4B will bring new hope and breakthroughs in the treatment of OSA comorbid with hypertension.

## 2 Materials and Methods

### 2.1 Animal Models

#### 2.1.1 Ethics Statement

All procedures complied with ARRIVE guidelines and were approved by the Institutional Animal Care and Use Committee at the Affiliated Yan’an Hospital of Kunming Medical University Kunming Hypertension Center.

#### 2.1.2 Animals

Specific pathogen‐free (SPF) male Sprague–Dawley rats (8 weeks old, 200 ± 20 g; Charles River Laboratories, Strain Code 400) were acclimatised for 7 days in individually ventilated cages (Tecniplast GM500) under controlled conditions: 22°C ± 1°C, 55% ± 5% humidity, 12‐h light/dark cycle (lights on 07:00), with ad libitum access to irradiated chow (LabDiet 5053) and autoclaved water.

### 2.2 Chronic Intermittent Hypoxia (CIH) Model

#### 2.2.1 Hypoxia System

Customised chambers (Biospherix ProOx C21) regulated via O_2_/N_2_ gas mixing (GasBlender 100, BioSpherix) with real‐time oxygen monitoring (MAX‐250 analyser, ±0.1% accuracy).

#### 2.2.2 Protocol

90‐s cycles (60‐s hypoxia: 6.5% ± 0.5% O_2_, 0.1% CO_2_; 30‐s reoxygenation: 21% O_2_) for 8 h/day (09:00–17:00), 7 days/week for 6 weeks. Control rats (*n* = 12/group) received normoxic exposure (21% O_2_) in identical chambers.

#### 2.2.3 Physiological Monitoring

Daily body weight (±0.1 g), food/water intake (±1 mL), and non‐invasive blood pressure (ALC‐NIBP, Shanghai Aikang) measured via tail‐cuff plethysmography (3 consecutive readings at 5‐min intervals). Systemic blood pressure was measured weekly using a non‐invasive tail‐cuff plethysmography system (Kent Scientific, USA) under conscious and restrained conditions. Three consecutive measurements were averaged for each time point.

#### 2.2.4 AAV1 Delivery

At Week 2, rats were anaesthetised with 2.5% isoflurane (1 L/min O_2_) and intratracheally injected with 1 × 10^11^ viral genomes (vg) of AAV1 encoding either shRNA targeting PDE4B (AAV1.shPDE4B; Hanbio, HB‐AAV‐025) or a control shRNA (AAV1.Luc; Hanbio, HB‐AAV‐018) in 50 μL sterile PBS (HyClone, SH30256.01) using a 24G catheter (BD Insyte). The AAV1 vectors also carried a GFP reporter gene under a separate promoter to allow visualisation and quantification of transduction efficiency in target tissues. Viral titre was validated by qPCR quantification (AAVpro Titration Kit, Takara, 6233) with a threshold cycle (Ct) value < 28. Post‐injection, chronic intermittent hypoxia (CIH) exposure was continued for 4 weeks. Knockdown efficiency and specificity were confirmed by Western blot and qPCR in cardiac and pulmonary tissues, with no significant off‐target effects observed on other PDE4 isoforms (e.g., PDE4A, PDE4C, PDE4D).

### 2.3 Lung Tissue Histology and Immunostaining

#### 2.3.1 Section Preparation

Paraffin sections: 4 μm sections were deparaffinised in xylene (3 × 5 min), rehydrated through graded ethanol, and antigen‐retrieved in citrate buffer (pH 6.0, 95°C, 20 min; Dako, S1699).

#### 2.3.2 Cryosections

10 μm sections fixed in 4% paraformaldehyde (Electron Microscopy Sciences, 15,710) for 15 min at 25°C.

### 2.4 Staining Protocols

#### 2.4.1 H&E/Masson's Trichrome

Automated staining (Leica ST5020) following manufacturer's protocols. Small pulmonary arteries (50–200 μm diameter) adjacent to terminal bronchioles were analyzed. Quantitative assessment of pulmonary vascular remodeling was performed on Masson's trichrome‐stained lung sections. Medial wall thickness was calculated as a percentage of media width relative to external vessel diameter. The muscularization status of small pulmonary arteries (< 50 μm in diameter) was classified as non‐, partially, or fully muscularized based on smooth muscle actin staining.

#### 2.4.2 Immunofluorescence

Blocked with 5% BSA (Sigma, A7906)/0.3% Triton X‐100 (Sigma, T9284) for 1 h, then incubated overnight at 4°C with primary antibodies: anti‐PDE4B (Abcam, ab170941, 1:200), anti‐α‐SMA (Abcam, ab7817, 1:500), anti‐Ki67 (Cell Signalling, 9129S, 1:400), anti‐cleaved Caspase‐3 (CST, 9664, 1:300). Secondary antibodies: Alexa Fluor 488/594 (Invitrogen, 1:1000). Nuclei counterstained with DAPI (5 μg/mL, Thermo, P36931).

#### 2.4.3 Imaging and Analysis

Slides scanned at 20× (Zeiss LSM 880, Zen 3.0 software). Quantification performed by two blinded investigators using ImageJ (NIH) with inter‐rater reliability (ICC > 0.85).

### 2.5 Assessment of Right Ventricular Systolic Pressure and Hypertrophy

#### 2.5.1 Right Ventricular Catheterization

Under 2.5% isoflurane anaesthesia, a heparinized (10 U/mL) 25G catheter (YPJ01H, Chengdu Biological Instruments) was percutaneously inserted into the right ventricle via subxiphoid approach. Pressure waveforms were recorded at a 1 kHz sampling rate (RM6240 system) over 10 consecutive cardiac cycles.

#### 2.5.2 Right Ventricular Hypertrophy (RVH)

Hearts excised, atria removed. RV free wall separated from left ventricle (LV) + septum (S) using microscissors (Fine Science Tools, 15000‐03). RV/(LV + S) mass ratio calculated (Mettler Toledo XS205, ±0.1 mg). Cardiomyocyte cross‐sectional area measured in FITC‐WGA (Invitrogen, W32466) stained sections (≥ 50 cells/animal, ImageJ).

### 2.6 Cell Culture and Hypoxia Modelling

#### 2.6.1 PASMCs

Human pulmonary artery smooth muscle cells (ScienCell, 3110) from 3 male donors (45–60 years) were authenticated by STR profiling and α‐SMA/vimentin positivity (flow cytometry > 95%). Cultured in DMEM (Gibco, 11,965,092) supplemented with 10% FBS (Gibco, 10437028), 1% penicillin–streptomycin (Gibco, 15140122) at 37°C/5% CO_2_. Used at passages 4–6.

#### 2.6.2 siRNA Transfection

At 70% confluence, cells were transfected with 50 nM siRNA (Hanbio) using Lipofectamine 3000 (Invitrogen, L3000001; 3 μL/well). Knockdown efficiency was confirmed by RT‐qPCR (TaqMan assays, Applied Biosystems) at 48 h (ΔΔCt method, GAPDH normalization). Sequences are in Table S1.

#### 2.6.3 Cyclic Hypoxia (CIH)

Cells in the hypoxia chamber (Biospherix C21) underwent 6 cycles/day of 35‐min hypoxia (1% O_2_, 5% CO_2_) → 25‐min normoxia (21% O_2_) for 72 h. Media were replaced every 24 h with pre‐equilibrated solutions. Normoxic controls were maintained at 21% O_2_.

### 2.7 Western Blot Analysis

#### 2.7.1 Sample Preparation

##### 2.7.1.1 Cell/Tissue Lysis

Cells or 30 mg frozen tissue were homogenised in RIPA lysis buffer (25 mM Tris–HCl pH 7.6, 150 mM NaCl, 1% NP‐40, 1% sodium deoxycholate, 0.1% SDS; Beyotime, P0013B) supplemented with protease/phosphatase inhibitors (Roche, 04693159001). Lysates were centrifuged at 13,000 × *g* for 30 min at 4°C.

##### 2.7.1.2 Protein Quantification

Supernatant protein concentrations were determined via BCA assay (Pierce, 23,225), with absorbance measured at 562 nm (SpectraMax M5, Molecular Devices).

##### 2.7.1.3 Electrophoresis and Transfer

Thirty micrograms total protein per lane was separated on 10% SDS‐PAGE gels (Bio‐Rad, 4561033) at 100 V for 2 h. Proteins were transferred to PVDF membranes (0.45 μm, Millipore, IPFL00010) using a semi‐dry system (Bio‐Rad Trans‐Blot Turbo) at 25 V for 30 min.

#### 2.7.2 Antibody Incubation

Membranes blocked with 5% non‐fat milk (Bio‐Rad, 1,706,404) in TBST (20 mM Tris, 150 mM NaCl, 0.1% Tween‐20) for 1 h at 25°C.

– Primary antibodies incubated overnight at 4°C with gentle shaking:
  - Anti‐PDE4B (1:1000, CST 72096).
  - Anti‐HK2 (1:1500, CST 2867S).
  - Anti‐PKM2 (1:1000, CST 4053S).
  - Anti‐HIF1α (1:800, Abcam ab179483).
  - Anti‐β‐actin (1:5000, ab8226) as a loading control. β‐actin is the preferred loading control, based on its known stability under hypoxic and oxidative stress conditions.
– HRP‐conjugated secondary antibodies (1:5000, CST 7076) incubated for 1 h at 25°C.

#### 2.7.3 Detection

Chemiluminescent signals developed with ECL Prime (GE Healthcare, RPN2232) and captured using Odyssey Fc Imager (LI‐COR). Band intensity quantified via Image Studio 5.2 (LI‐COR), normalized to α‐tubulin. Three biological replicates per group.

### 2.8 Reverse Transcription‐Quantitative PCR (RT‐qPCR)

#### 2.8.1 RNA Isolation and QC

Total RNA extracted with TRIzol (Takara, 9108) followed by DNase I treatment (Thermo, EN0521). RNA purity assessed (A260/A280: 1.8–2.0; A260/A230 > 2.0) using NanoDrop ND‐1000. RNA integrity confirmed by 1% agarose gel electrophoresis (RIN > 7).

#### 2.8.2 cDNA Synthesis

One microgram RNA reverse‐transcribed using PrimeScript RT Master Mix (Takara, RR036A) in 20 μL reactions: 42°C for 30 min, 85°C for 5 s (Thermo Cycler 9700).

#### 2.8.3 qPCR Amplification

Reactions performed in triplicate with SYBR Green Premix (Takara, RR420A) on QuantStudio 5 (Applied Biosystems): 95°C 30 s → 40 cycles of 95°C 5 s, 60°C 30 s. Melt curve analysis (60°C–95°C) confirmed specificity.

#### 2.8.4 Primers

Designed via NCBI Primer‐BLAST (span exon‐exon junctions). Sequences in Table S2. Data analyzed by 2^−ΔΔCt^ method using GAPDH (F:5′‐GGAGCGAGATCCCTCCAAAAT‐3′, R:5′‐GGCTGTTGTCATACTTCTCATGG‐3′) as reference.

### 2.9 Cell Proliferation and Apoptosis Assays

#### 2.9.1 CCK‐8 Assay

PASMCs (5 × 10^3^ cells/well) seeded in 96‐well plates. At 0/24/48/72 h, 10 μL CCK‐8 (Dojindo, CK04) was added per well. Incubated for 2 h at 37°C. Absorbance was measured at 450 nm (630 nm reference) using a Microplate Reader (BioTek Synergy H1).

#### 2.9.2 EdU Proliferation Assay

Cells treated with 10 μM EdU (Beyotime, C0071S) for 2 h. Fixed with 4% PFA, permeabilized (0.5% Triton X‐100), stained with Apollo 567 (30 min, dark). Nuclei counterstained with Hoechst 33342 (5 μg/mL). Images acquired via Nikon Eclipse Ti2 (10× objective). Proliferation rate = (EdU + cells/total cells) × 100%.

#### 2.9.3 Caspase‐3 Apoptosis Assay

Cells stained with FITC‐DEVD‐FMK (Beyotime, C1073S) for 1 h at 37°C. Counterstained with PI (5 μg/mL). Fluorescence quantified by flow cytometry (BD FACSCanto II). Apoptotic cells defined as FITC+/PI‐.

### 2.10 Transwell Migration Assay

Upper chambers (8 μm pores, Corning 3422) coated with 50 μL Matrigel (1:8 dilution; BD Biosciences, 354,234). PASMCs (2 × 10^4^ cells/well) in serum‐free DMEM were added to the upper chamber. The lower chamber was filled with 600 μL DMEM +20% FBS.

After 24 h, non‐migrated cells were removed with a cotton swab. Migrated cells were fixed (4% PFA), stained (0.5% crystal violet, 10 min). Five random fields per well were imaged (Nikon TS2, 10×). Cell counts were analysed via ImageJ.

### 2.11 Oxidative Stress Parameter Analysis

#### 2.11.1 Sample Preparation

Cells lysed in RIPA buffer, centrifuged (12,000 × g, 10 min). Supernatant protein adjusted to 2 mg/mL.

#### 2.11.2 Assays

##### 2.11.2.1 MDA

Lipid peroxidation measured via thiobarbituric acid reaction (Beyotime, S0131S). Absorbance at 532 nm.

##### 2.11.2.2 SOD

Activity determined using the WST‐8 method (Beyotime, S0101S). One unit = 50% inhibition of superoxide reduction.

##### 2.11.2.3 GSH/GSSG

Total glutathione and GSSG quantified via enzymatic recycling (Beyotime, S0053). GSH = Total glutathione −2 ×GSSG.

#### 2.11.3 Calculations

– MDA (nmol/mg protein) = (Sample OD – Blank OD)/Standard curve slope.
– SOD activity (%) = [(Acontrol − Asample)/Acontrol] × 100.
– GSH/GSSG ratio = (Total GSH – 2 × GSSG)/GSSG.

### 2.12 Detection of Mitochondrial Reactive Oxygen Species and Membrane Potential

#### 2.12.1 JC‐1 Mitochondrial Membrane Potential Assay

##### 2.12.1.1 Protocol

PASMCs (2 × 10^4^ cells/well in 24‐well plates) were incubated with 2 μM JC‐1 (Beyotime, C2003S) in serum‐free DMEM at 37°C for 20 min. Cells were rinsed twice with JC‐1 staining buffer and imaged immediately.

##### 2.12.1.2 Imaging

Fluorescence was captured using an Olympus FV3000 confocal microscope with dual‐channel detection:

– J‐aggregates (polarised mitochondria): Ex/Em = 585/590 nm (red channel).
– J‐monomers (depolarized mitochondria): Ex/Em = 485/535 nm (green channel).

##### 2.12.1.3 Quantification

Red/green fluorescence intensity ratio was calculated using ImageJ (≥ 50 cells/group). Negative controls included cells treated with 10 μM CCCP (mitochondrial uncoupler, Sigma, C2759) for 30 min.

#### 2.12.2 MitoSOX Red mtROS Detection

##### 2.12.2.1 Staining

Cells were loaded with 5 μM MitoSOX Red (Invitrogen, M36009) in HBSS for 30 min at 37°C, followed by 1 μM MitoTracker Green (Invitrogen, M36008) for 15 min.

##### 2.12.2.2 Confocal Imaging

Z‐stack images (60× oil objective, 2 μm step size) were acquired under identical settings. mtROS intensity was normalized to MitoTracker Green signal (mitochondrial mass).

##### 2.12.2.3 Specificity Controls

Pre‐treatment with 10 μM MitoTEMPO (mtROS scavenger, Sigma, SML0737) for 2 h confirmed signal specificity (Pearson's colocalization coefficient > 0.85).

### 2.13 Transmission Electron Microscopy (TEM)

#### 2.13.1 Sample Preparation

Cells were fixed in 2.5% glutaraldehyde (Electron Microscopy Sciences, 16,220) in 0.1 M phosphate buffer (pH 7.4) for 2 h at 25°C. Post‐wash with 0.1 M cacodylate buffer, cells were treated with 2% osmium tetroxide (EMS, 19150) + 1.5% potassium ferrocyanide (Sigma, P8131) in the same buffer for 2 h at 4°C. Samples were immersed in 2% uranyl acetate (EMS, 22400) overnight at 4°C.

#### 2.13.2 Embedding and Sectioning

Graded ethanol series (30%, 50%, 70%, 90%, 100%) for 10 min each. EPON 812 resin (EMS, 14120) polymerization at 65°C for 48 h. Ultrathin sections (70 ± 5 nm) cut using a Leica UC7 ultramicrotome with a diamond knife (Diatome, Ultra 45°).

#### 2.13.3 Imaging and Analysis

Sections were post‐stained with Reynolds lead citrate for 5 min. Images acquired at 80 kV using a JEOL JEM‐1400 TEM equipped with a XAROSA CMOS camera (8 k × 8 k resolution). Mitochondrial cristae density was quantified via ImageJ (≥ 20 mitochondria/cell, 10 cells/group).

### 2.14 Glucose Uptake and Metabolic Intermediate Assays

#### 2.14.1 Glucose Uptake

##### 2.14.1.1 Kit

Glucose Uptake Colorimetric Assay (Biovision, K686‐100).

##### 2.14.1.2 Protocol

1 × 10^4^ cells/well were serum‐starved (4 h), incubated with 10 mM 2‐DG (Sigma, D8375) for 20 min. Lysates were neutralized with 0.1 M Tris–HCl (pH 8.0). Absorbance at 412 nm was measured (BioTek Synergy H1).

##### 2.14.1.3 Normalisation

Data normalised to total protein (BCA assay, Pierce 23225).

#### 2.14.2 Pyruvate/Lactate/ATP Quantification

##### 2.14.2.1 Pyruvate

Pyruvate Colorimetric Kit (Biovision, K609‐100; *λ* = 570 nm).

##### 2.14.2.2 Lactate

Lactate Assay Kit (Biovision, K607‐100; *λ* = 450 nm).

##### 2.14.2.3 ATP

ATP Colorimetric Kit (Biovision, K354‐100; *λ* = 570 nm).

##### 2.14.2.4 Standards

Freshly prepared pyruvate (0–10 nmol/well) and ATP (0–20 nmol/well) standard curves.

### 2.15 Glycolytic Enzyme Activity Assays

#### 2.15.1 Sample Preparation

##### 2.15.1.1 Cell Lysis

– HK/PFK/ALDO: 5 × 10^5^ cells in 50 mM Tris–HCl (pH 7.5), 1% Triton X‐100, protease inhibitors.
– PK/LDH: 5 × 10^5^ cells in ice‐cold PBS + 0.1% digitonin.

#### 2.15.2 Activity Measurement

##### 2.15.2.1 Hexokinase (HK)

HK Activity Kit (Biovision, K789‐100). Reaction monitored at 340 nm (NADPH generation).

##### 2.15.2.2 Phosphofructokinase (PFK)

PFK Activity Kit (Biovision, K763‐100). Coupled NADH oxidation at 340 nm.

##### 2.15.2.3 Aldolase (ALDO)

ALDO Activity Kit (Biovision, K665‐100). Absorbance at 240 nm (fructose‐1,6‐bisphosphate cleavage).

##### 2.15.2.4 Pyruvate Kinase (PK)

PK Activity Kit (Biovision, K709‐100). ADP generation via NADH‐coupled reaction (λ = 340 nm).

##### 2.15.2.5 LDH

LDH Activity Kit (Biovision, K730‐500). Lactate‐to‐pyruvate conversion (*λ* = 450 nm).

##### 2.15.2.6 Data Expression

Activities expressed as mU/mg protein (1 U = 1 μmol product/min).

### 2.16 Extracellular Acidification and Oxygen Consumption Rate Assays

#### 2.16.1 Seahorse XF Protocol

##### 2.16.1.1 Cell Seeding

1 × 10^4^ PASMCs/well in XFe 96 plate. Pre‐equilibrated in Seahorse XF DMEM (Agilent, 103575‐100) pH 7.4 for 1 h.

##### 2.16.1.2 Stress Test Conditions

– ECAR (Glycolysis):
  1. Basal: 10 mM glucose (Agilent, 103,577–100).
  2. Glycolytic Capacity: 1 μM oligomycin (Sigma, 75351).
  3. Glycolytic Reserve: 50 mM 2‐DG.
– OCR (Mitochondrial Respiration):
  1. Basal Respiration: Untreated.
  2. ATP‐linked: 1 μM oligomycin.
  3. Maximal Respiration: 1.5 μM FCCP (Sigma, C2920).
  4. Non‐mitochondrial: 0.5 μM rotenone +0.5 μM antimycin A (Sigma, R8875/A8674).

##### 2.16.1.3 Normalisation

Data normalised to cell count via CyQUANT NF assay (Invitrogen, C35006).

##### 2.16.1.4 Analysis

Seahorse Wave 2.6.1 software with baseline correction (3 cycles pre‐injection).

#### 2.16.2 Plasmid Generation

##### 2.16.2.1 Vector Construction

The parental plasmid pGFP‐C1‐FUS (Addgene) was digested with NheI‐HF (20 U/μg, NEB, R3131S) and SacI (20 U/μg, NEB, R0156S) at 37°C for 3 h. The **RFP cDNA** (Clontech, #632504) was ligated into the linearized backbone using T4 DNA ligase (NEB, M0202S) at 16°C overnight.

##### 2.16.2.2 Site‐Directed Mutagenesis

The pRFP‐C1‐FUS‐Y6/296F mutant was generated using the QuikChange II XL Kit (Agilent, 200,521). Primers (Sigma‐Aldrich):

– FUS‐Y6F‐F: 5′‐GCTTCAAACGACT**T**TAC‐3′ (mutation site underlined).
– FUS‐Y296F‐R: 5′‐GTAATCAGCCACAGA**T**TC‐3′.

##### 2.16.2.3 Vector Purification

Plasmids were isolated using the Plasmid Plus Midi Kit (Qiagen, 12,945) and quantified via Nanodrop (A260/A280 > 1.8).

### 2.17 Chromatin Immunoprecipitation (ChIP)

#### 2.17.1 Crosslinking and Sonication

Cells (1 × 10^8^) were fixed with 1% formaldehyde (Thermo, 28,906) for 10 min at 25°C, quenched with 125 mM glycine.

#### 2.17.2 Sonication

Chromatin was sheared using a QSonica Q700 (amplitude 40%, 10 s on/15 s off, 8 min total) with a 2 mm probe. Fragment size (200–500 bp) was confirmed by agarose gel (1.5%).

#### 2.17.3 Immunoprecipitation

Pre‐cleared chromatin (150 μg) was incubated with 5 μg anti‐H3K18la (PTM‐1406RM, Jingjie) or IgG control (Abcam, ab172730) overnight at 4°C.

#### 2.17.4 Bead Capture

Protein A/G magnetic beads (Millipore, 16‐663) were washed 3× with ChIP buffer (50 mM HEPES pH 7.5, 150 mM NaCl, 1% Triton X‐100) and incubated with chromatin‐antibody complexes for 6 h.

#### 2.17.5 DNA Recovery and Sequencing

Crosslinks were reversed with 200 mM NaCl at 65°C overnight. DNA was purified using ChIP DNA Clean & Concentrator (Zymo, D5205). Libraries were prepared with NEBNext Ultra II DNA Library Prep Kit (NEB, E7645S). Sequencing was performed on Illumina NovaSeq 6000 (150 bp paired‐end, 40 M reads/sample).

### 2.18 Luciferase Reporter Assay

#### 2.18.1 Transfection and Measurement

Cells (2 × 10^4^/well in 24‐well plates) were co‐transfected with 500 ng reporter plasmid (pGL4.10[luc2], Promega, E6651) and 50 ng pRL‐TK (Renilla control, Promega, E2241) using Lipofectamine 3000 (Invitrogen, L3000001).

#### 2.18.2 Dual‐Luciferase Assay

Forty‐eight hours post‐transfection, cells lysed with Passive Lysis Buffer (Promega, E1941). Firefly/Renilla luciferase activities measured sequentially using GloMax Navigator (Promega) with a 5 s integration time.

### 2.19 Immunoprecipitation (IP)

#### 2.19.1 Procedure

Cells lysed in NP‐40 buffer (50 mM Tris pH 7.4, 150 mM NaCl, 1% NP‐40, protease inhibitors). Lysates (500 μg) pre‐cleared with Protein G agarose (GE, 17–0618‐01) for 1 h. Pre‐cleared lysates incubated with 2 μg anti‐phosphotyrosine (4G10, Millipore, 05‐321) overnight at 4°C. Immune complexes captured with Protein G agarose (2 h, 4°C). Beads boiled in 2× Laemmli buffer (Bio‐Rad, 1,610,737) at 95°C for 10 min. Eluates analyzed by Western blot (10% gel, 100 V 90 min).

### 2.20 Cellular Immunofluorescent Staining

#### 2.20.1 Protocol

Cells fixed with 4% PFA (Electron Microscopy Sciences, 15,710) for 15 min, permeabilized with 0.5% Triton X‐100 (Sigma, T9284) in PBS for 10 min. 1% BSA (Sigma, A7906)/PBST for 1 h. Primary antibody (anti‐FUS, Sigma, HPA008784, 1:200) incubated overnight at 4°C. Alexa Fluor 488 goat anti‐rabbit IgG (Invitrogen, A‐11008, 1:1000) applied for 1 h. Nuclei counterstained with DAPI (5 μg/mL). Images acquired on Nikon Eclipse Ti2 (40× air objective, NIS‐Elements 5.30 software).

### 2.21 Statistical Analysis

All statistical analyses were conducted using GraphPad Prism 9.0 software (GraphPad Software LLC, San Diego, CA, USA). Data are presented as the mean ± standard deviation (SD) derived from at least three independent experiments, each performed in triplicate. Prior to conducting parametric tests, normality was assessed using the Shapiro–Wilk test for datasets with *n* < 50 or the Kolmogorov–Smirnov test for larger datasets. Homogeneity of variance was also evaluated to ensure appropriate use of parametric methods. Comparisons between two groups were analysed using an unpaired, two‐tailed Student's *t*‐test when data met the assumptions of normality and equal variances. For comparisons among more than two groups, one‐way analysis of variance (ANOVA) followed by Tukey's post hoc test was employed to adjust for multiple comparisons. When data failed to meet the assumptions required for parametric testing, non‐parametric alternatives were applied: the Mann–Whitney U test was used for two‐group comparisons, and the Kruskal‐Wallis test with Dunn's post hoc correction was used for multi‐group comparisons. A P‐value threshold of less than 0.05 (*p* < 0.05) was considered statistically significant throughout this study. Additionally, effect sizes were calculated to quantify the magnitude of differences observed between groups, providing a more comprehensive understanding beyond statistical significance alone.

## 3 Results

### 3.1 PDE4B Expression Is Elevated in the OSA Combined With Pulmonary Hypertension Model, Promoting PASMCs Reprogramming

**FIGURE 1:**
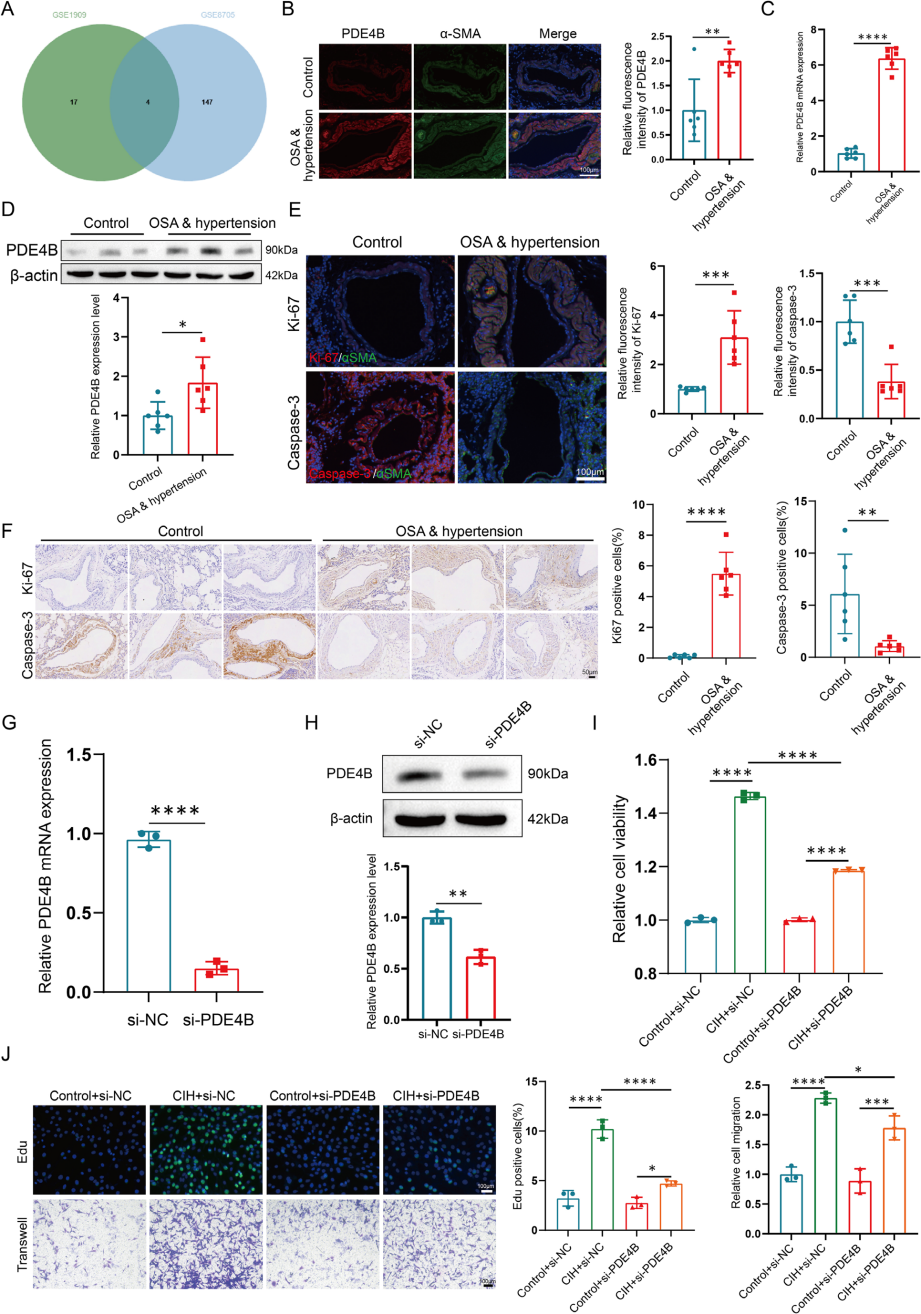
PDE4B Expression is Elevated in the OSA Combined with Hypertension Model, Promoting PASMC Reprogramming. (A) Integration of gene expression profiles from two independent datasets (GSE1909: Chronic hypoxia‐induced pulmonary hypertension; GSE8705: Obstructive sleep apnea). Both datasets were analysed to identify common pathways involved in pulmonary vascular remodelling. Venn diagram showing overlap between significantly dysregulated genes identified in each condition. (B) Representative images and relative fluorescence intensities of pulmonary arteries from normal and OSA rats, double immunostained for PDE4B (red) and α‐SMA (green), highlighting the smooth muscle layer. Scale bar: 100 μm. Data are presented as mean ± SD (*n* = 6 per group). Statistical analysis was performed using an unpaired Student's *t*‐test. **p < 0.01, ****p < 0.0001. (C, D) Quantification of PDE4B mRNA (C) and protein (D) expression levels in lung homogenates from normal and OSA rats. Data are presented as mean ± SD (*n* = 6 per group). Statistical analysis was performed using an unpaired Student's *t*‐test. *p < 0.05, ****p < 0.0001. (E, F) Evaluation of proliferation (Ki67; E) and apoptosis (Caspase‐3; F) in PASMCs from normal and OSA rats, with representative images of Ki67 (top) and Caspase‐3 (bottom) staining. Scale bar: 100 μm. Data are presented as mean ± SD (*n* = 6 per group). Statistical analysis was performed using an unpaired Student's *t*‐test. **p < 0.01, ***p < 0.001, ****p < 0.0001. (G, H) Analysis of PDE4B mRNA (G) and protein (H) expression in PASMCs treated with si‐NC or si‐PDE4B. Data are presented as mean ± SD (*n* = 3 per group). Statistical analysis was performed using an unpaired Student's *t*‐test. **p < 0.01, ****p < 0.0001. (I) Cell viability measured using a CCK‐8 kit across four groups. Data are presented as mean ± SD (*n* = 3 per group). Statistical analysis was performed using an unpaired Student's *t*‐test. ****p < 0.0001. (J) Assessment of proliferation (EdU, green; top) and migration (bottom) in PASMCs treated with or without si‐PDE4B under normal or CIH conditions. Scale bar: 100 μm. Data are presented as mean ± SD (*n* = 3 per group). Statistical analysis was performed using an unpaired Student's *t*‐test. *p < 0.05, ****p < 0.0001.

To investigate the molecular mechanisms underlying the pathogenesis of OSA combined with hypertension, we utilized two publicly available datasets, GSE1909 (related to chronic hypoxia‐induced pulmonary hypertension) and GSE8705 (involving OSA), to identify common pathways and potential therapeutic targets. Despite the different etiologies, both conditions share overlapping pathophysiological mechanisms, particularly in terms of vascular remodeling and inflammation. Our analysis revealed that PDE4B, a crucial enzyme involved in signal transduction, was significantly associated with OSA combined with hypertension (Figure 1A). To validate these findings, we examined PDE4B expression in samples from rats with OSA‐induced hypertension. The results indicated a significant upregulation of PDE4B, particularly in PASMCs (Figure 1B–D). Furthermore, we assessed proliferation and apoptosis in PASMCs derived from OSA patients. Compared to controls, PASMCs from OSA patients exhibited increased proliferation and reduced apoptosis rates (Figure 1E,F).

To elucidate the role of PDE4B in PASMCs reprogramming under conditions mimicking OSA‐induced hypertension, we established a chronic intermittent hypoxia (CIH) model in vitro and silenced PDE4B using siRNA (Figure 1G,H). Notably, CIH exposure led to enhanced PASMC activity, whereas PDE4B silencing significantly mitigated this effect, resulting in improved cell proliferation and migration (Figure 1I,J). Collectively, these data indicate that elevated PDE4B expression contributes to PASMCs reprogramming in the context of OSA combined with hypertension.

### 3.2 Knockout of PDE4B Improves OSA Combined With Hypertension Phenotype

**FIGURE 2:**
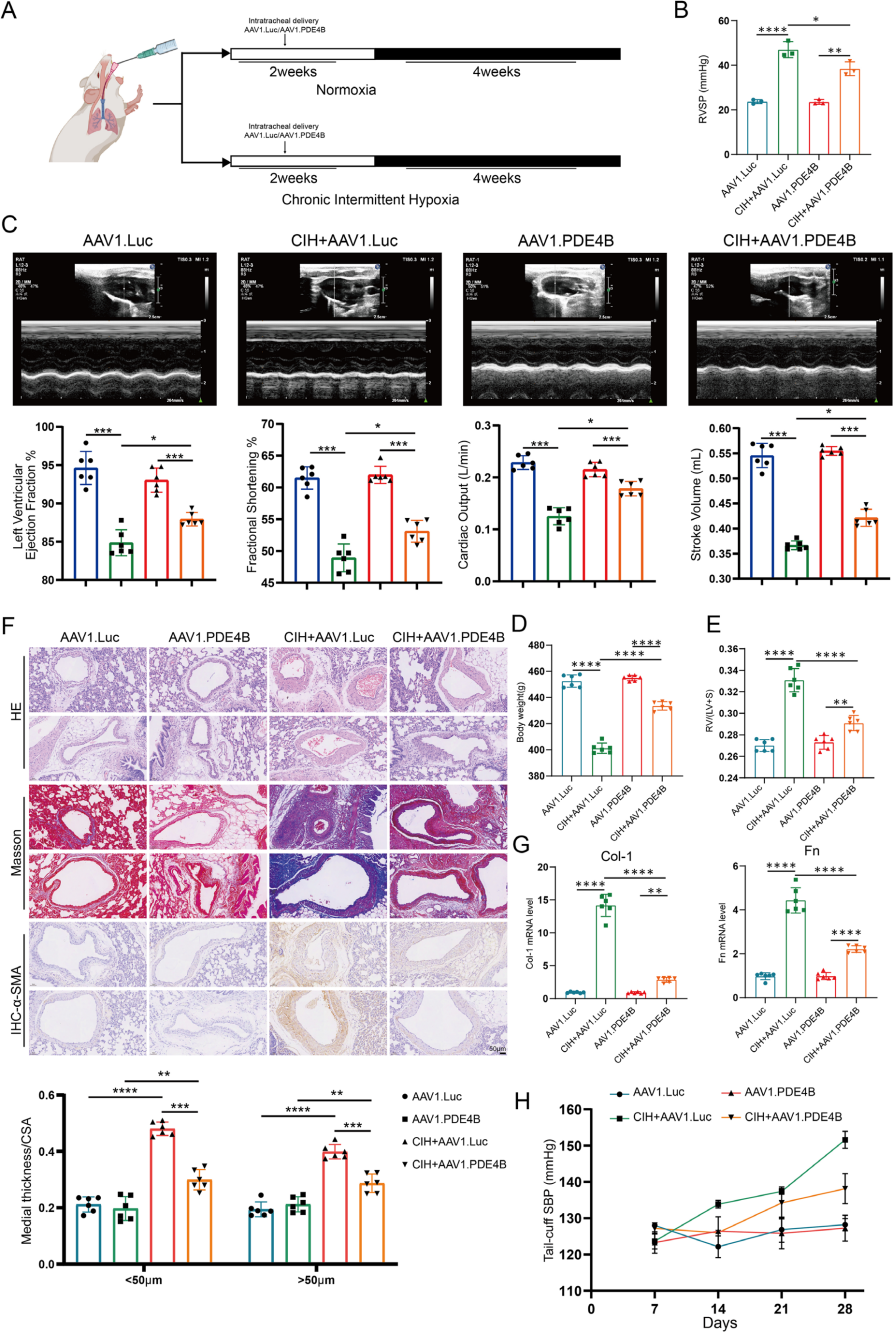
Knockout of PDE4B Improves OSA Combined with Hypertension Phenotype. (A) Schematic representation of the experimental design for AAV1.shPDE4B treatment. (B) Waveform diagrams and quantitative analysis of RVSP (mmHg). Data are presented as mean ± SD (*n* = 3 per group). Statistical analysis was performed using an unpaired Student's *t*‐test. *p < 0.05, **p < 0.01, ****p < 0.0001. (C) Echocardiographic analyses of CIH rats. Data are presented as mean ± SD (*n* = 6 per group). (D) Body weight of rats. Data are presented as mean ± SD (*n* = 6 per group). Statistical analysis was performed using one‐way ANOVA followed by Tukey's post hoc test. ****p < 0.0001. (E) Fulton index (RV/(LV + S)) of rats. Data are presented as mean ± SD (*n* = 6 per group). Statistical analysis was performed using one‐way ANOVA followed by Tukey's post hoc test. **p < 0.01, ****p < 0.0001. (F) Representative images of pulmonary arteries stained with H&E, Masson, and α‐SMA. Scale bar: 50 μm. Data are presented as mean ± SD (*n* = 6 per group). Statistical analysis was performed using one‐way ANOVA followed by Tukey's post hoc test. **p < 0.01, ***p < 0.001, ****p < 0.0001. (G) mRNA expression levels of fibrosis‐associated proteins (Col‐1 and Fibronectin) in rat lungs. Data are presented as mean ± SD (*n* = 6 per group). Statistical analysis was performed using one‐way ANOVA followed by Tukey's post hoc test. **p < 0.01, ****p < 0.0001. (H) Measured systolic blood pressure in rats. Data are presented as mean ± SD (*n* = 6 per group). **p < 0.01, ****p < 0.0001.

To further evaluate the therapeutic potential of PDE4B knockout in an animal model of OSA combined with hypertension, rats were subjected to CIH for 2 weeks followed by intratracheal administration of either AAV1.PDE4B or AAV1.Luc as a control for four additional weeks (Figure 2A). Successful transfection of AAV1.PDE4B into lung tissues was confirmed, leading to a significant reduction in PDE4B expression levels (Figure S1A–C). As hypothesized, PDE4B knockout effectively ameliorated pulmonary hypertension in CIH‐treated rats. Specifically, compared to the AAV1.Luc group, animals treated with AAV1.PDE4B exhibited markedly lower right ventricular systolic pressure (RVSP) and reduced distal pulmonary artery remodelling, along with partial restoration of body weight (Figure 2B–E). We have now included echocardiographic analyses of CIH rats (Figure 2C), demonstrating significant right ventricular dysfunction (reduced TAPSE, RVFAC) and left ventricular hypertrophy (increased LVMI), consistent with combined PH and systemic hypertension. Additionally, PDE4B knockout attenuated CIH‐induced right ventricular hypertrophy and decreased the expression of lung fibrosis‐associated proteins around the perivascular regions (Figures 2F,G and S1D). Medial wall thickness was significantly increased in the OSA + hypertension group compared to control, and this increase was attenuated by PDE4B knockdown. As expected, chronic intermittent hypoxia induced a progressive increase in systemic systolic blood pressure, which was significantly reduced by PDE4B knockdown (Figure 2H). These findings collectively suggest that PDE4B knockout can substantially improve the phenotype associated with OSA combined with hypertension.

### 3.3 The High Expression of PDE4B Is Associated With Oxidative Stress and Mitochondrial Damage in PASMCs Within the OSA Combined With Hypertension

**FIGURE 3:**
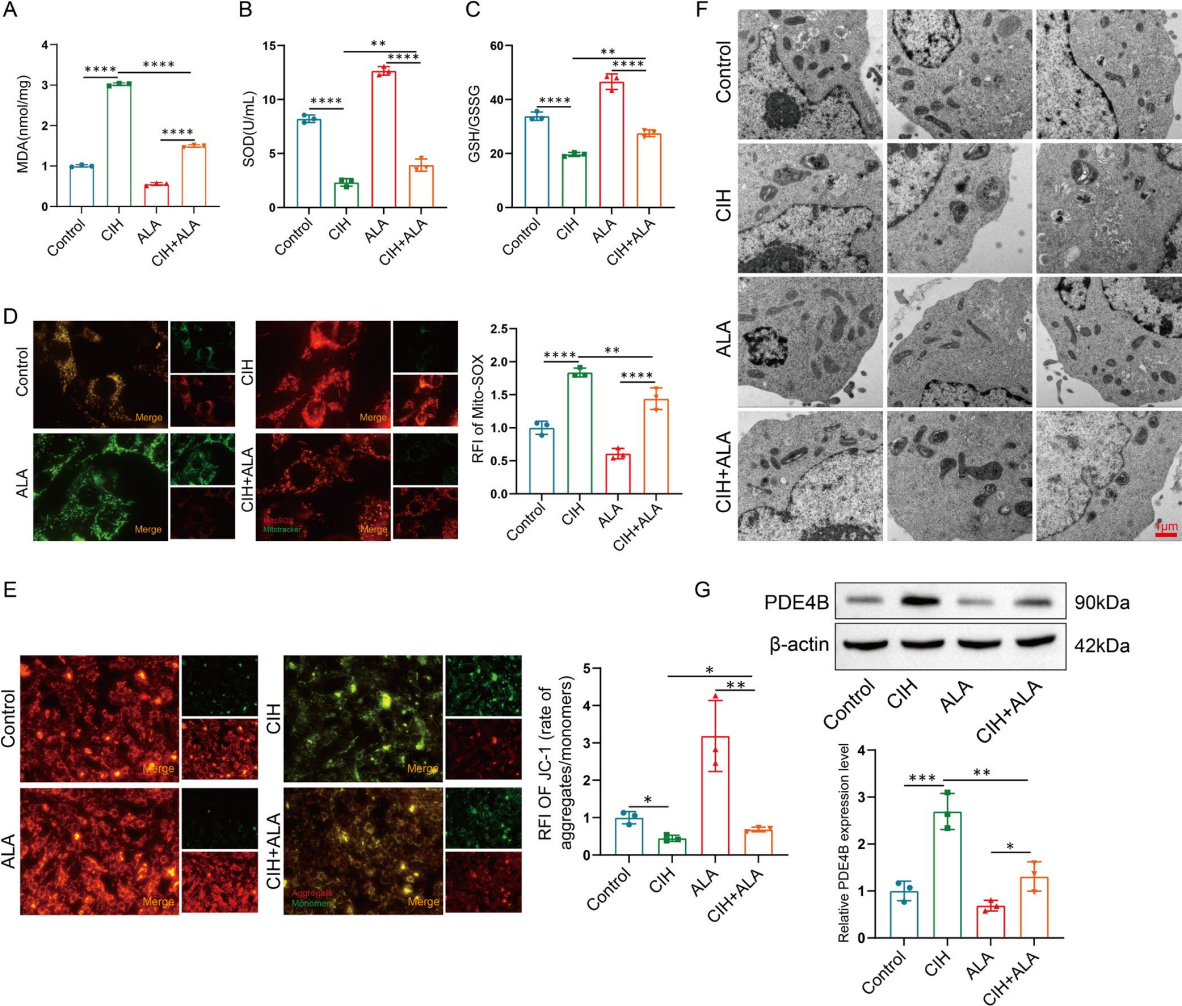
High expression of PDE4B is associated with oxidative stress and mitochondrial damage in PASMCs within the OSA combined with hypertension. (A–C) Levels of MDA (A), SOD (B), and GSH/GSSG (C) in PASMCs were detected. Data are presented as mean ± SD (*n* = 3 per group). Statistical analysis was performed using an unpaired Student's *t*‐test. **p < 0.01, ****p < 0.0001. (D) Representative images and relative fluorescence intensities of mtROS in PASMCs assessed using MitoSOX, with mitochondria located by MitoTracker green. Scale bar: 5 μm. Data are presented as mean ± SD (*n* = 3 per group). Statistical analysis was performed using an unpaired Student's *t*‐test. **p < 0.01, ****p < 0.0001. (E) Representative images and relative fluorescence intensities of mitochondrial membrane potential in PASMCs assessed using JC‐1 kit, where aggregates/monomer (red/green fluorescence) indicates mitochondrial membrane potential depolarization. Scale bar: 10 μm. Data are presented as mean ± SD (*n* = 3 per group). Statistical analysis was performed using an unpaired Student's *t*‐test. *p < 0.05, **p < 0.01. (F) Representative TEM images of mitochondria in PASMCs. Scale bar: 1 μm. (G) Analysis of PDE4B protein expression and quantification in PASMCs. Data are presented as mean ± SD (*n* = 3 per group). Statistical analysis was performed using an unpaired Student's *t*‐test. *p < 0.05, **p < 0.01, ***p < 0.001.

To evaluate the impact of oxidative stress on pulmonary artery smooth muscle cells (PASMCs) in the context of OSA combined with hypertension, we measured levels of malondialdehyde (MDA), superoxide dismutase (SOD), and glutathione/glutathione disulfide (GSH/GSSG). MDA serves as a biomarker for lipid peroxidation, reflecting oxidative stress levels, whereas SOD and GSH/GSSG are indicators of cellular antioxidant capacity [16, 17]. Our findings demonstrated that CIH treatment led to increased MDA concentrations and decreased levels of SOD and GSH/GSSG in PASMCs, indicating elevated oxidative stress. Treatment with alpha‐lipoic acid (ALA), an oxidative stress inhibitor, reversed these effects, reducing MDA and increasing SOD and GSH/GSSG levels (Figure 3A–C).

Subsequently, mitochondrial damage in PASMCs was assessed post‐CIH exposure. We observed heightened mitochondrial reactive oxygen species (mtROS) production and reduced mitochondrial membrane potential, alongside notable mitochondrial swelling and cristae disappearance. These alterations were partially mitigated by ALA treatment (Figure 3D–F). To further elucidate the relationship between oxidative stress, mitochondrial damage, and PDE4B expression in this model, we analyzed PDE4B levels following CIH and ALA treatments. Elevated PDE4B expression after CIH was significantly attenuated by ALA administration (Figure 3G). Collectively, these data suggest that PDE4B upregulation in PASMCs is positively correlated with oxidative stress and mitochondrial dysfunction.

### 3.4 Oxidative Stress and Mitochondrial Damage Lead to Abnormal Glycolysis in PASMCs

**FIGURE 4:**
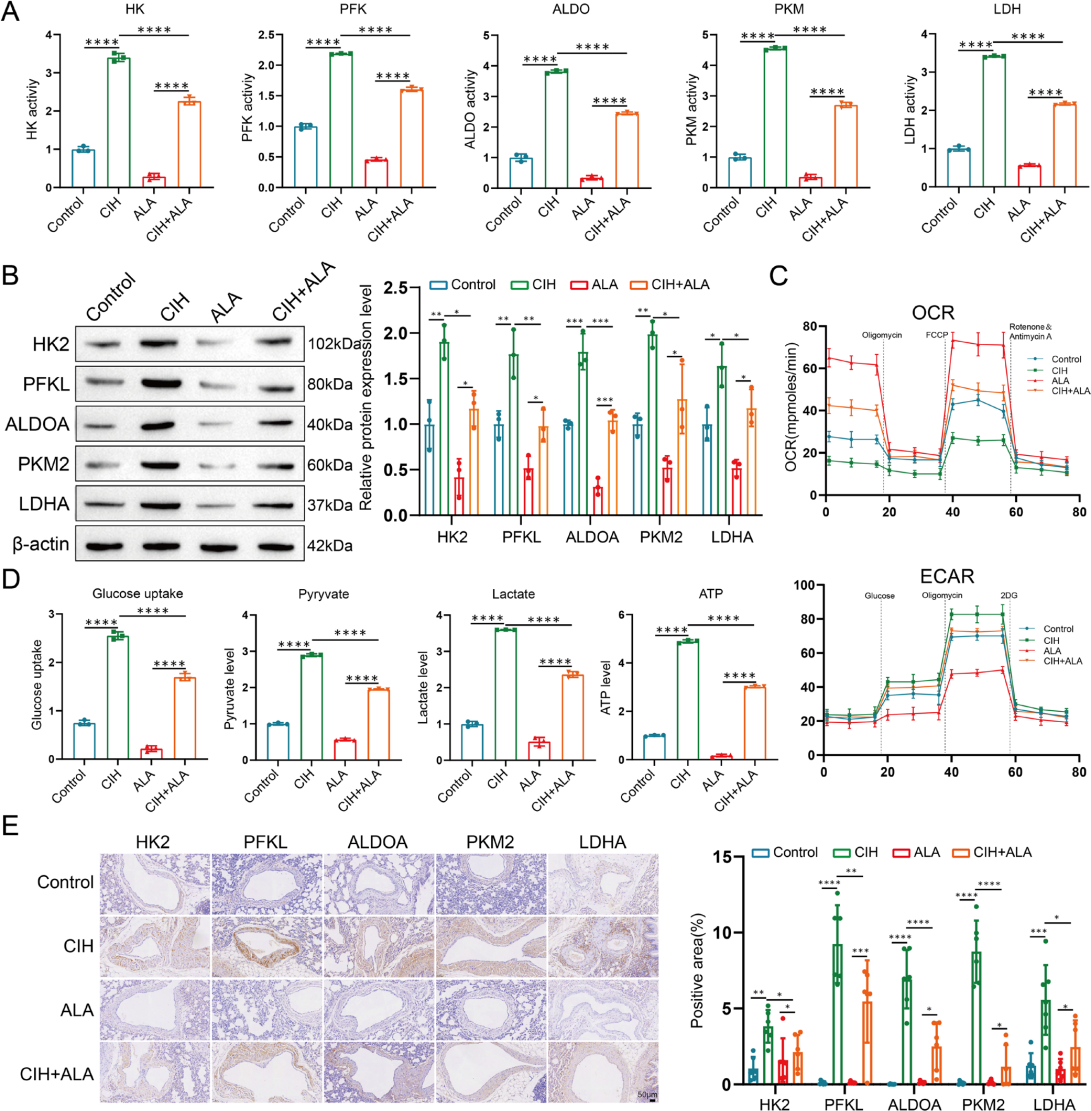
Oxidative stress and mitochondrial damage lead to abnormal glycolysis in PASMCs. (A) Activities of HK, PFK, ALDO, PKM, and LDH in PASMCs. Data are presented as mean ± SD (*n* = 3 per group). Statistical analysis was performed using an unpaired Student's *t*‐test. ****p < 0.0001. (B) Protein expression and quantification of HK2, PFKL, ALDOA, PKM2, and LDHA in PASMCs. Data are presented as mean ± SD (*n* = 3 per group). Statistical analysis was performed using an unpaired Student's *t*‐test. *p < 0.05, **p < 0.01, ***p < 0.001. (C) ECAR and OCR measurements in PASMCs. Data are presented as mean ± SD (*n* = 3 per group). Statistical analysis was performed using an unpaired Student's *t*‐test. *p < 0.05, **p < 0.01, ***p < 0.001. (D) Glucose uptake, pyruvate level, lactate production, and ATP level in PASMCs. Data are presented as mean ± SD (*n* = 3 per group). Statistical analysis was performed using an unpaired Student's *t*‐test. ****p < 0.0001. (E) Representative images and semiquantitative analysis of pulmonary arteries stained with HK2, PFKL, ALDOA, PKM2, and LDHA in rats. Scale bar: 50 μm. Data are presented as mean ± SD (*n* = 6 per group). Statistical analysis was performed using one‐way ANOVA followed by Tukey's post hoc test. *p < 0.05, **p < 0.01, ***p < 0.001, ****p < 0.0001.

Given the significant enhancement of glycolysis under conditions of cellular hypoxia or stress to meet rapid energy demands [18], we examined the glycolytic phenotype in PASMCs exposed to CIH and treated with ALA. CIH treatment promoted the activities of key glycolytic enzymes including HK, PFK, ALDO, PKM2, and LDH. ALA treatment counteracted these effects (Figure 4A). Additionally, CIH increased the expression of HK2, PFKL, ALDOA, PKM2, and LDHA, which was also reversed by ALA (Figure 4B). Oxygen consumption rate (OCR), indicative of mitochondrial oxidative phosphorylation, was reduced in CIH‐exposed cells, while extracellular acidification rate (ECAR), reflecting glycolytic activity, was increased; both effects were ameliorated by ALA (Figure 4C). Moreover, CIH enhanced glucose uptake, pyruvate levels, lactate production, and ATP levels in PASMCs, all of which were normalized by ALA treatment (Figure 4D). In vivo studies mirrored these findings, showing increased expression of HK2, PFKL, ALDOA, PKM2, and LDHA in rats subjected to CIH, with reductions noted following ALA administration (Figure 4E). These results collectively indicate that oxidative stress and mitochondrial damage contribute to aberrant glycolysis in PASMCs.

### 3.5 Lactate Accumulation Induces Histone Lactylation Promoting the Transcription and Expression of PDE4B**

**FIGURE 5:**
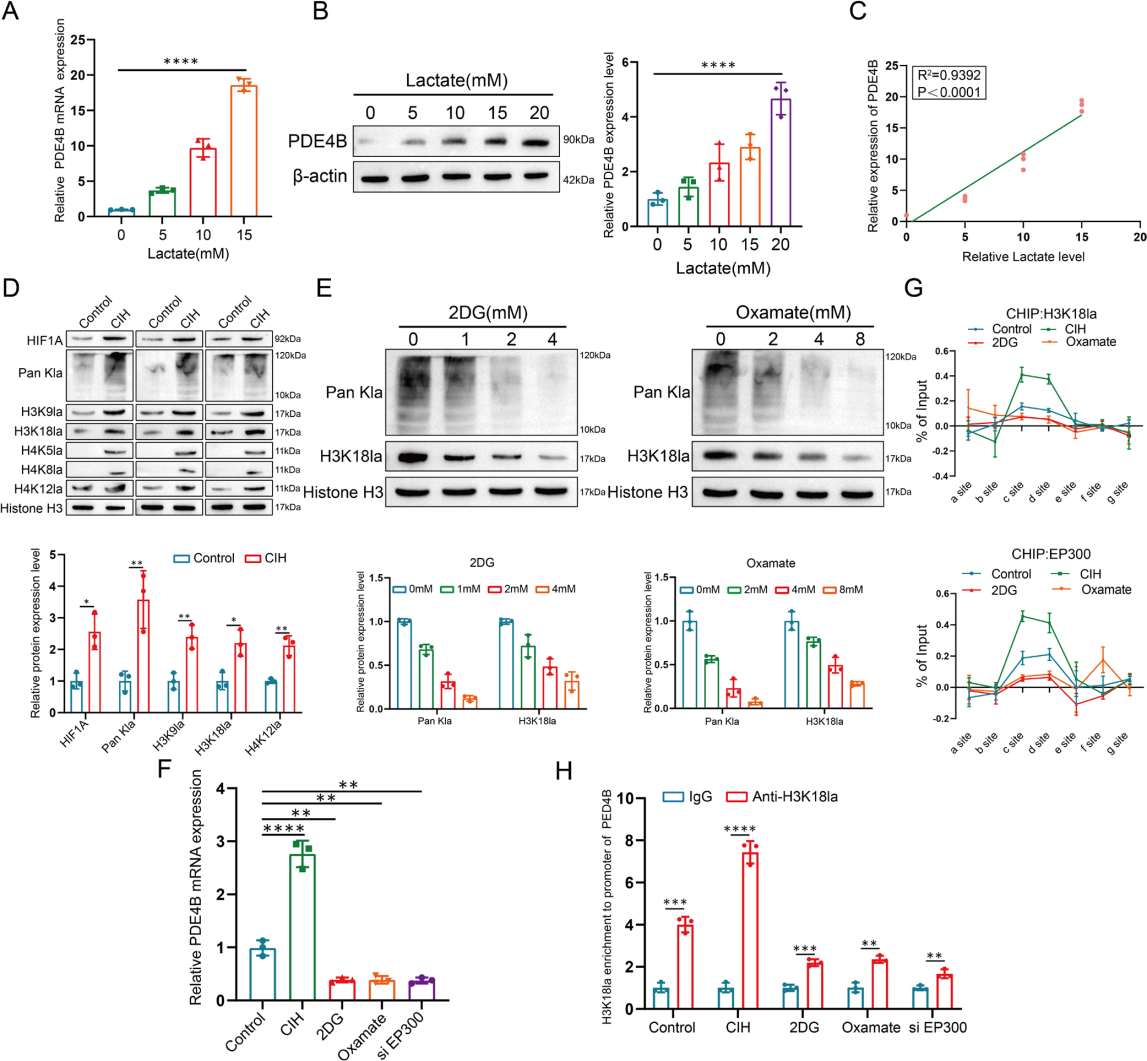
Lactate accumulation induces histone lactylation promoting the transcription and expression of PDE4B. (A, B) Analysis of PDE4B mRNA (A) and protein (B) expression in PASMCs cultured in different concentrations of lactate. Data are presented as mean ± SD (*n* = 3 per group). Statistical analysis was performed using one‐way ANOVA followed by Tukey's post hoc test. ****p < 0.0001. (C) Correlation between PDE4B expression levels and lactylation levels in PASMCs. Pearson correlation coefficient (*r*) and *p* value are indicated. (D) Analysis of histone lactylation protein expression and quantification in PASMCs. Data are presented as mean ± SD (*n* = 3 per group). Statistical analysis was performed using one‐way ANOVA followed by Tukey's post hoc test. *p < 0.05, **p < 0.01. (E) Expression and quantification of Pan Kla and H3K18la proteins in PASMCs treated with varying concentrations of 2‐DG or oxamate. Data are presented as mean ± SD (*n* = 3 per group). Statistical analysis was performed using one‐way ANOVA followed by Tukey's post hoc test. ****p < 0.0001. (F) RT‐PCR analysis of PDE4B mRNA expression levels in PASMCs. Data are presented as mean ± SD (*n* = 3 per group). Statistical analysis was performed using one‐way ANOVA followed by Tukey's post hoc test. **p < 0.01, ****p < 0.0001. (G–H) ChIP‐qPCR assays measuring H3K18la and EP300 status at the PDE4B genomic region (G) and promoter occupancy (H) in PASMCs. Data are presented as mean ± SD (*n* = 3 per group). Statistical analysis was performed using one‐way ANOVA followed by Tukey's post hoc test. **p < 0.01, ***p < 0.001.

Lactate, a major end product of glycolysis, has been shown to promote histone lactylation through its accumulation [19, 20]. Given our previous findings that PDE4B expression is associated with oxidative stress, we aimed to elucidate the regulatory role of histone lactylation induced by lactate in the gene expression of PDE4B under oxidative conditions. Our initial experiments demonstrated that increasing concentrations of lactate led to elevated PDE4B expression in PASMCs (Figure 5A–C).

To further explore the biological functions of histone lactylation in PASMCs, Western blot analysis was performed using antibodies against various forms of histone lactylation. This revealed a baseline level of H3K18la under normal conditions, which significantly increased following CIH treatment (Figure 5D). To block intracellular lactate production, glycolytic inhibitors (2‐deoxy‐D‐glucose [2‐DG] and oxamate) were employed. These inhibitors effectively reduced global lactylation levels and specifically H3K18la in a dose‐dependent manner (Figure 5E).

Previous studies have indicated that EP300 (E1A binding protein p300) regulates histone lactylation levels [21, 22]. Interestingly, our results showed that glycolytic inhibitors did not affect EP300 protein expression levels (Figure S2A). However, both glycolytic inhibitors and siRNA targeting EP300 led to decreased mRNA levels of PDE4B (Figures 5F and S2B,C). Chromatin immunoprecipitation followed by PCR (ChIP‐PCR) revealed significant enrichment of H3K18la and EP300 at the PDE4B promoter regions after CIH treatment, effects that were mitigated by glycolytic inhibitors (Figure 5G,H). Additionally, the enrichment of H3K27ac at the PDE4B promoter region remained unaffected by CIH and glycolytic inhibitor treatments or si‐EP300 (Figure S2D), as did the stability of PDE4B mRNA (Figure S2E). These findings indicate that the transcription and expression of PDE4B in PASMCs are positively regulated by H3K18la.

### 3.6 PDE4B Influences the Reprogramming of PASMCs via AGT

**FIGURE 6:**
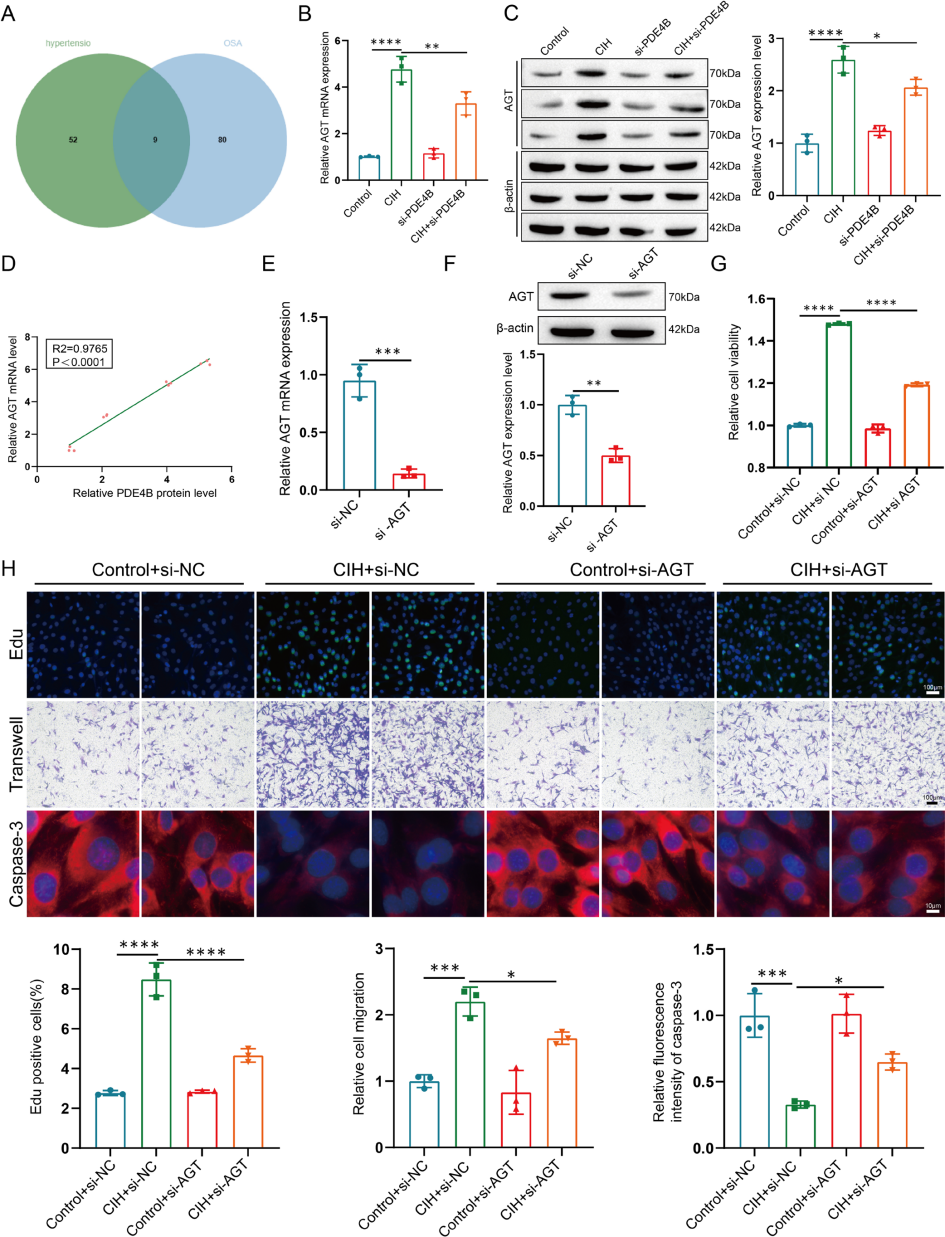
PDE4B influences the reprogramming of PASMCs via AGT. (A) Venn diagram of regulating gene datasets related to hypertension and OSA. (B, C) Analysis of AGT mRNA (B) and protein (C) expression levels in PASMCs. Data are presented as mean ± SD (*n* = 3 per group). Statistical analysis was performed using one‐way ANOVA followed by Tukey's post hoc test. *p < 0.05, **p < 0.01, ****p < 0.0001. (D) Correlation between mRNA expression levels of AGT and protein expression levels of PDE4B in PASMCs. Pearson correlation coefficient (*r*) and *p*‐value are indicated. (E, F) Analysis of AGT mRNA (E) and protein (F) expression in PASMCs treated with si‐NC or si‐AGT. Data are presented as mean ± SD (*n* = 3 per group). Statistical analysis was performed using an unpaired Student's *t*‐test. **p < 0.01, ***p < 0.001. (G) Cell viability measurement using a CCK‐8 kit. Data are presented as mean ± SD (*n* = 3 per group). Statistical analysis was performed using one‐way ANOVA followed by Tukey's post hoc test. ****p < 0.0001. (H) Evaluation of proliferation (EdU, green; top), migration (middle), and apoptosis (Caspase, red; bottom) in PASMCs treated or not treated with si‐AGT under normal or CIH conditions. Scale bar: 100 μm. Data are presented as mean ± SD (*n* = 3 per group). Statistical analysis was performed using one‐way ANOVA followed by Tukey's post hoc test. *p < 0.05, ***p < 0.001, ****p < 0.0001.

To better understand how PDE4B affects the reprogramming of PASMCs in OSA comorbid with hypertension, we identified genes regulating hypertension and OSA from the GeneCards database (Figure 6A). Among these, angiotensinogen (AGT) emerged as a key regulator of blood pressure, influencing vascular tone and remodelling [23]. We hypothesized that PDE4B influences OSA comorbid with hypertension through regulation of AGT.

To test this hypothesis, we first examined AGT expression in PASMCs. Results showed that AGT expression was significantly upregulated following CIH treatment, an effect that was reversed upon silencing PDE4B (Figure 6B,C). Correlation analysis confirmed a relationship between PDE4B and AGT expression (Figure 6D). Since AGT can be upregulated by PDE4B, we next investigated its functional role in PASMCs. Successful knockdown of AGT using siRNA (Figure 6E,F) was followed by CCK‐8 assays, which demonstrated improved cell viability, an effect that was rescued by AGT silencing (Figure 6G). Furthermore, AGT knockdown enhanced PASMC proliferation and migration while increasing apoptosis rates following CIH treatment (Figure 6H). Collectively, these data suggest that PDE4B influences the reprogramming of PASMCs in the context of OSA comorbid with hypertension by regulating AGT.

### 3.7 PDE4B Influences the Localization and Function of FUS Through Its Phosphorylation

**FIGURE 7:**
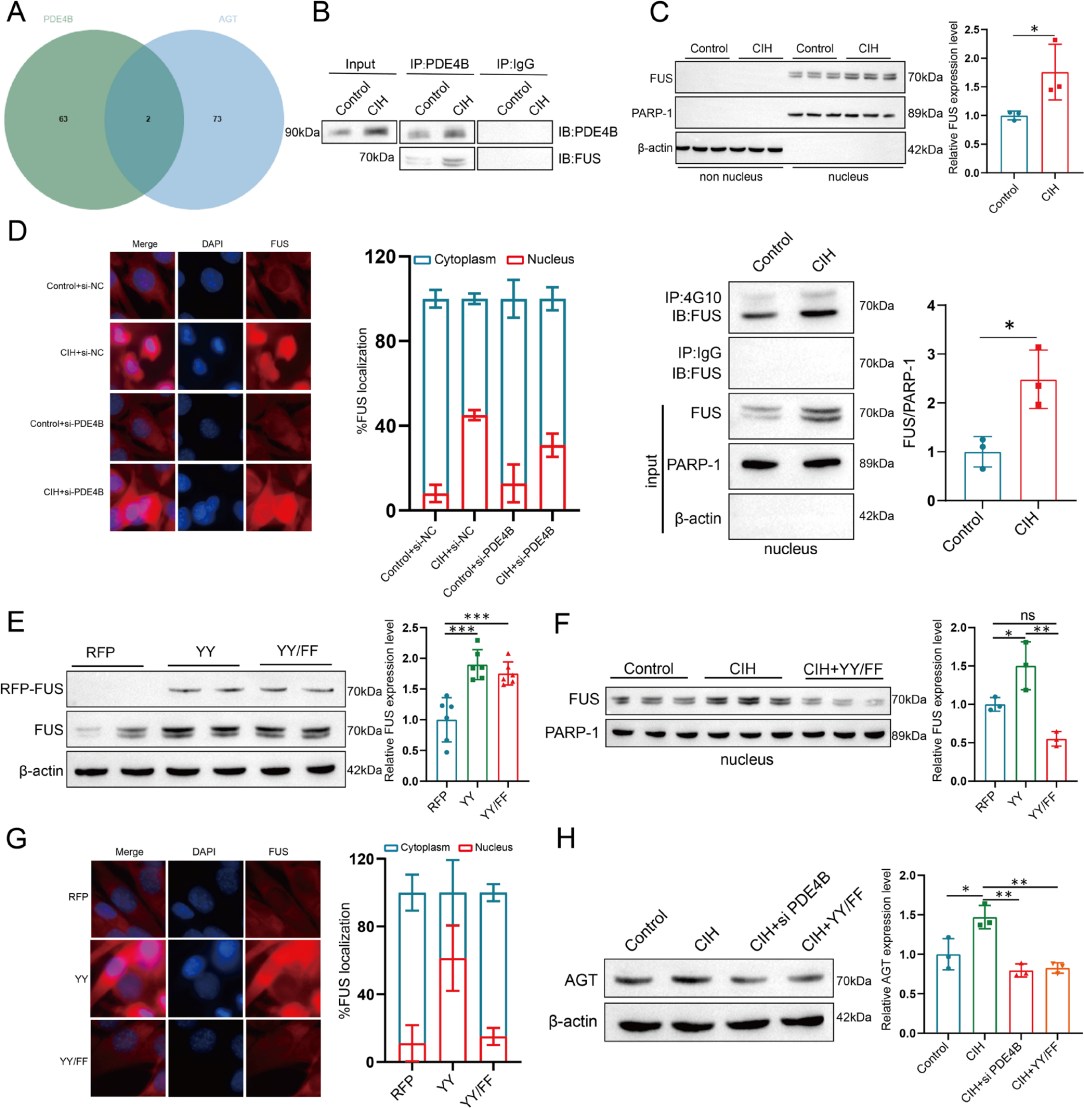
PDE4B Influences the Localization and Function of FUS Through Its Phosphorylation. (A) Venn diagram of interacting proteins between PDE4B and AGT. (B) Western blot analysis of cell lysates from PASMCs with control or CIH treatment, immunoprecipitated with anti‐PDE4B antibody or IgG control, showing levels of PDE4B and FUS. (C) Analysis of FUS protein expression in non‐nuclear and nuclear fractions from PASMCs with control or CIH treatment, with nuclear fractions immunoprecipitated using anti‐phosphotyrosine antibody or IgG control. (D) Representative images and semiquantitative analysis of FUS (green) localization in PASMCs. Scale bar: 10 μm. Data are presented as mean ± SD (*n* = 3 per group). Statistical analysis was performed using one‐way ANOVA followed by Tukey's post hoc test. (E) Transient transfection of PASMCs with pRFP‐C1 empty vector (RFP), pRFP‐C1 fused to WT FUS (YY), or FUS construct with mutated tyrosines 6 and 296 (YY/FF), followed by Western blot analysis for RFP‐FUS levels. ***p < 0.001. (F–G) Analysis of FUS protein expression and quantification in nuclear fractions of PASMCs, with representative images (G). Scale bar: 10 μm. Data are presented as mean ± SD (*n* = 3 per group). Statistical analysis was performed using one‐way ANOVA followed by Tukey's post hoc test. *p < 0.05, **p < 0.01. (H) Analysis of AGT protein expression and quantification in PASMCs. Data are presented as mean ± SD (*n* = 3 per group). Statistical analysis was performed using one‐way ANOVA followed by Tukey's post hoc test. *p < 0.05, **p < 0.01.

To understand how PDE4B regulates AGT gene transcription and translation in PASMCs, we initially predicted potential PDE4B‐interacting proteins using the BioGRID database. Recent studies have highlighted the role of RNA‐binding proteins (RBPs) in vascular smooth muscle cell function and cardiovascular disease pathogenesis [24, 25]. Utilizing the ENCORI database, we identified RBPs interacting with AGT and found that FUS may play a critical role in PDE4B‐mediated regulation of AGT expression (Figures 7A and S3A,B).

Immunoprecipitation assays using anti‐PDE4B or IgG control antibodies followed by Western blotting for FUS revealed an interaction between PDE4B and FUS in both control and CIH‐treated PASMCs, though this interaction was more pronounced in CIH‐treated cells (Figure 7B). Further analysis indicated increased nuclear localization of FUS in CIH‐treated PASMCs compared to controls (Figure 7C). Immunoprecipitation of nuclear fractions using antiphosphotyrosine antibodies showed enhanced tyrosine phosphorylation of nuclear FUS in CIH‐treated cells (Figure 7C). Additionally, immunofluorescence staining confirmed increased nuclear FUS levels post‐CIH treatment, which were rescued by PDE4B silencing (Figure 7D), suggesting a close relationship between PDE4B activity, FUS phosphorylation, and its nuclear localization.

To elucidate the role of PDE4B in regulating AGT expression through FUS, we transiently transfected PASMCs with RFP‐FUS (wild‐type FUS) or RFP‐FUS‐Y6/296F (mutant FUS with Y‐to‐F substitutions at phosphorylation sites Y6 and Y296) (Figures 7E and S3C). Analysis of nuclear FUS levels showed a significant elevation in WT FUS‐expressing cells, whereas mutant FUS did not show significant changes compared to controls (Figure 7F). Immunofluorescence staining supported these findings (Figure 7G). Moreover, AGT expression was significantly reduced in cells with silenced PDE4B or expressing mutant FUS compared to CIH‐treated cells (Figure 7H). These results suggest that PDE4B promotes nuclear translocation of FUS via its phosphorylation.

### 3.8 FUS Binds to the AGT Promoter and Promotes Its Transcription

**FIGURE 8:**
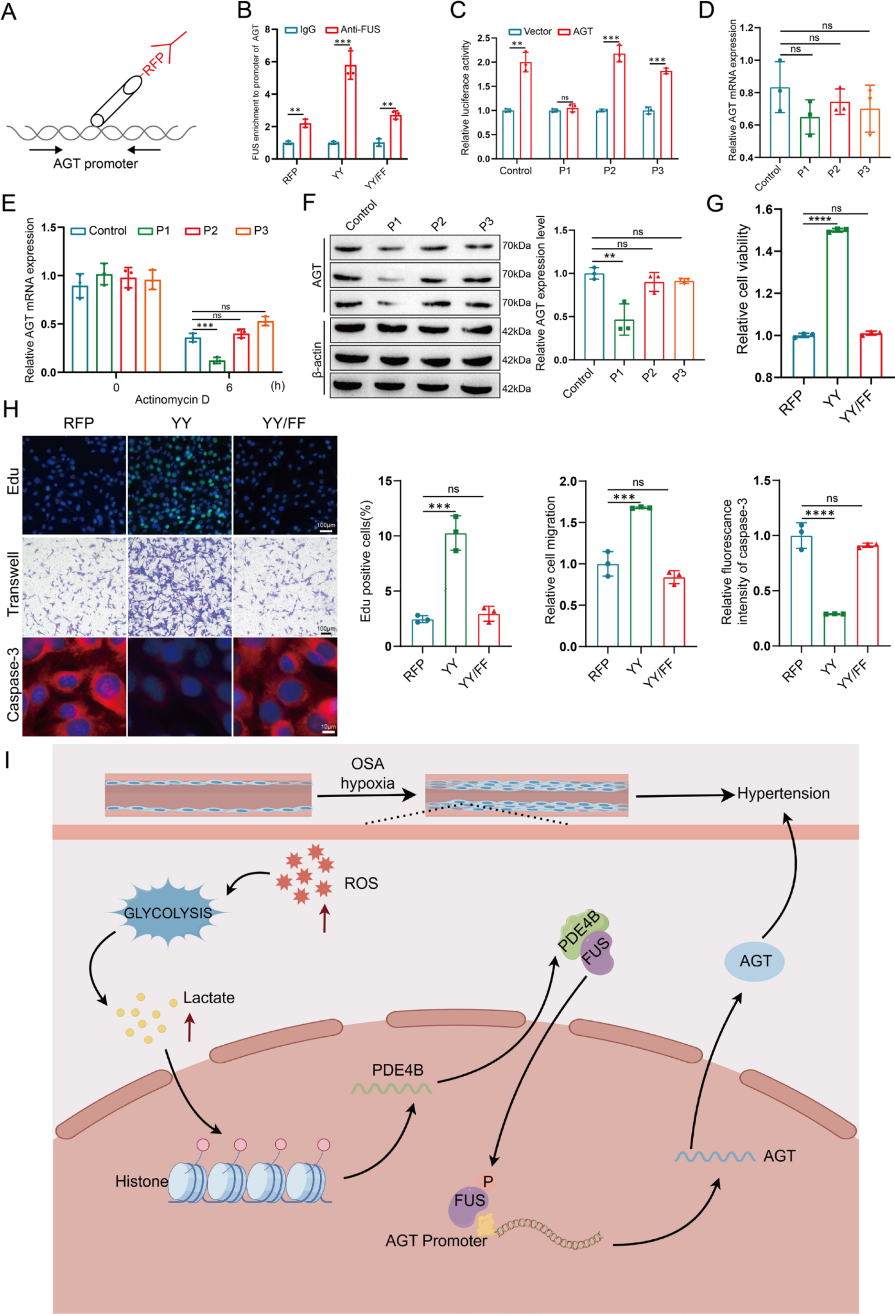
FUS binds to the AGT promoter and promotes its transcription. (A) Overview of ChIP assays performed in PASMCs expressing RFP, YY, or YY/FF constructs. (B) ChIP‐qPCR analysis of FUS occupancy at the AGT promoter in PASMCs. Data are presented as mean ± SD (*n* = 3 per group). Statistical analysis was performed using one‐way ANOVA followed by Tukey's post hoc test. **p < 0.01, ***p < 0.001. (C) Luciferase reporter assay measuring relative luciferase activity of AGT after mutating the FUS binding site. Data are presented as mean ± SD (*n* = 3 per group). Statistical analysis was performed using one‐way ANOVA followed by Tukey's post hoc test. **p < 0.01, ***p < 0.001. (D) Analysis of AGT mRNA expression levels in PASMCs after mutating the FUS binding site. Data are presented as mean ± SD (*n* = 3 per group). Statistical analysis was performed using one‐way ANOVA followed by Tukey's post hoc test. Ns = no significance. (E) RT‐PCR analysis of AGT mRNA stability in PASMCs, with Actinomycin D used to block transcription. Data are presented as mean ± SD (*n* = 3 per group). Statistical analysis was performed using two‐way ANOVA followed by Bonferroni post hoc test. Ns = no significance, ***p < 0.001. (F) Analysis of AGT protein expression and quantification in PASMCs after mutating the FUS binding site. Data are presented as mean ± SD (*n* = 3 per group). Statistical analysis was performed using one‐way ANOVA followed by Tukey's post hoc test. **p < 0.01, ns = no significance. (G) Cell viability measurement using a CCK‐8 kit. Data are presented as mean ± SD (*n* = 3 per group). Statistical analysis was performed using one‐way ANOVA followed by Tukey's post hoc test. Ns = no significance, ***p < 0.001. (H) Evaluation of proliferation (EdU, green; top), migration (middle), and apoptosis (Caspase, red; bottom) in PASMCs transiently transfected with RFP, YY, or YY/FF. Scale bar: 100 μm. Data are presented as mean ± SD (*n* = 3 per group). Statistical analysis was performed using one‐way ANOVA followed by Tukey's post hoc test. Ns = no significance, ***p < 0.001. (I) Schematic diagram summarising the study findings: Obstructive sleep apnea‐induced oxidative stress triggers epigenetic modifications that activate the PDE4B‐FUS‐AGT signalling pathway leading to hypertension.

Given our findings on the nuclear localization of FUS affecting AGT protein expression, and considering FUS as an RBP for AGT, we hypothesized that FUS influences AGT transcription. Based on the presence of several FUS‐responsive elements within the bidirectional promoter of AGT (Figure S4A), we investigated whether FUS binds to the AGT promoter. Nuclei were immunoprecipitated using RFP‐TRAP beads, and the precipitated DNA was amplified using primers targeting a FUS binding motif in the AGT promoter (Figure 8A). ChIP‐PCR analysis demonstrated significantly increased FUS binding to the AGT promoter region in cells transfected with WT RFP‐FUS compared to those transfected with the RFP vector control or RFP‐FUS‐Y6/296F (Figure 8B).

Further analysis using the ATtRACT website predicted three putative FUS binding motifs (P1–P3) (Figure S4B). ChIP‐qPCR confirmed that the P1 site within the AGT promoter region is the primary binding site for FUS, rather than P2 or P3 (Figure 8C). Mutation of the P1 site did not reduce AGT mRNA expression but decreased mRNA stability and protein levels of AGT (Figure 8D–F). Collectively, these data indicate that the P1 site of FUS binds to the AGT promoter to promote its transcription and translation.

Finally, to investigate the role of FUS in PASMCs, CCK‐8 assays were performed, showing increased cell viability in WT FUS‐expressing cells and decreased viability in mutant FUS‐expressing cells compared to normal PASMCs (Figure 8G). Enhanced proliferation and migration, along with increased apoptosis rates, were observed in cells expressing mutant FUS (Figure 8H). Consistent with our in vitro findings, AGT and Ang II expression was upregulated in lung tissues of CIH‐exposed rats, which was attenuated by PDE4B knockdown, further supporting the involvement of the PDE4B‐FUS‐AGT pathway in vivo (Figure S5A,B). In summary, our findings suggest that FUS translocates into the nucleus and binds to the AGT promoter, potentially promoting its transcription and thereby influencing pulmonary artery smooth muscle cell (PASMC) reprogramming. Oxidative stress induced by chronic intermittent hypoxia (CIH), a key feature of OSA, may trigger epigenetic modifications that activate the PDE4B‐FUS‐AGT signalling axis. These observations identify this pathway as a potential regulatory mechanism in CIH‐associated hypertension, warranting further investigation to elucidate its functional role and therapeutic relevance (Figure 8I).

## 4 Discussion

In this study, we elucidated that oxidative stress in PASMCs induced by OSA leads to aberrant glycolysis, which subsequently induces histone lactylation. This process activates the PDE4B/FUS/AGT signalling pathway, thereby triggering hypertension. This discovery not only provides crucial insights into the molecular mechanisms underlying this comorbidity but also offers a novel perspective on therapeutic approaches. In the future, PDE4B inhibitors may serve as a promising therapeutic strategy to alleviate OSA‐associated hypertension.

PDE4B, a member of the phosphodiesterase family, primarily catalyzes the breakdown of cAMP, regulating intracellular cAMP levels and subsequently influencing various cellular signaling pathways [26, 27]. While GSE1909 focuses on chronic hypoxia‐induced pulmonary hypertension and GSE8705 is related to OSA, both diseases are characterized by abnormal vascular remodeling and inflammatory responses. By integrating these datasets, we aimed to uncover shared molecular pathways that could underlie the development of pulmonary hypertension in diverse clinical settings. Our research found that under the pathological conditions of OSA combined with hypertension, PDE4B expression is significantly elevated, suggesting that PDE4B may be involved in the pathogenesis of this disease. PASMCs play a crucial role in OSA combined with hypertension. The abnormal proliferation, migration, and enhanced contractility of these cells are key factors in the development of pulmonary arterial hypertension and hypertension [28, 29]. Our study confirmed that the knockout of PDE4B inhibited the viability, proliferation, and migration of PASMCs under the pathological conditions of OSA combined with hypertension, suggesting that elevated PDE4B expression may contribute to the development of the hypertensive phenotype in these patients. Furthermore, we observed that knocking out PDE4B significantly improved the phenotype of OSA combined with hypertension, demonstrating the central role of PDE4B in this disease. Currently, PDE4B inhibitors have already been applied in diseases such as pulmonary fibrosis, alcoholic liver disease, and multiple sclerosis [27, 30, 31], which provides a solid foundation for exploring the application of PDE4B in OSA comorbid with hypertension.

In OSA combined with hypertension, PASMCs are chronically exposed to cycles of intermittent hypoxia and reoxygenation, which readily trigger oxidative stress, leading to the massive production and accumulation of oxidative substances such as ROS. Our research found that under the pathological conditions of OSA combined with hypertension, oxidative stress in PASMCs is enhanced, manifested by increased MDA expression and decreased SOD and GSH/GSSG levels, and this effect is rescued by oxidative stress inhibitors. Mitochondria, the primary victims of oxidative stress [32, 33], suffered significant damage in PASMCs under the pathological conditions of OSA combined with hypertension, including elevated mROS, decreased mitochondrial membrane potential, swelling, and other pathological changes. This not only directly impacts the energy metabolism of PASMCs but may also promote cell apoptosis through mechanisms such as the release of apoptotic factors, further exacerbating the dysfunction of PASMCs. PDE4B, as one of the factors promoting the development of pulmonary vascular remodelling and pulmonary arterial hypertension, its high expression in OSA combined with hypertension may be related to the occurrence of oxidative stress. Our study also found that oxidative stress inhibitors rescued the high expression of PDE4B under the pathological conditions of OSA combined with hypertension, confirming the correlation between PDE4B, oxidative stress, and mitochondrial damage.

Our findings reveal that OSA‐induced oxidative stress activates the PDE4B/FUS/AGT axis in PASMCs, driving pulmonary vascular remodelling and PH. Importantly, OSA also promotes systemic hypertension through dual mechanisms. CIH‐triggered sympathetic overactivity and renal RAS upregulation elevate peripheral resistance, evidenced by increased systolic blood pressure and left ventricular hypertrophy in CIH rats. Mitochondrial dysfunction and glycolytic shift in systemic vasculature mirror PASMC changes, suggesting PDE4B contributes to systemic hypertension—supported by attenuated blood pressure upon PDE4B knockdown. Critically, CIH induced biventricular pathology: right ventricular dilation and diastolic dysfunction, confirming cardiopulmonary coupling in OSA. These data establish OSA as a unified driver of PH and systemic hypertension, with PDE4B inhibition offering a dual therapeutic strategy.

Under the dual blows of oxidative stress and mitochondrial damage, cells are often forced to seek alternative energy sources, thus shifting towards glycolysis. We found that under the pathological conditions of OSA combined with hypertension, the oxidative respiratory capacity of PASMCs decreased, while the glycolytic capacity increased, manifesting as increased activity of glycolytic enzymes and increased expression of related proteins and products. Lactate is an important metabolite of glycolysis, and our study also observed a concentration‐dependent increase in PDE4B expression in PASMCs with lactate, further confirming the regulatory role of oxidative stress on PDE4B. In recent years, the multifunctionality of lactate in cell biology has gradually attracted attention, particularly the histone lactylation modifications induced by lactate accumulation, which act as a novel epigenetic regulatory mechanism playing a critical role in various biological processes [9, 34]. Studies have shown that lactylation modifications can occur on multiple histones, such as H3 and H4, and these modifications can directly affect the transcriptional levels of target genes [28, 35]. Our studies indicate that under the pathological conditions of OSA combined with hypertension, the level of histone lactylation in PASMCs is elevated, and glycolytic inhibitors can inhibit the expression of PDE4B and the enrichment level of H3K18la on the PDE4B promoter. These results suggest that under the pathological conditions of OSA combined with hypertension, PASMCs experience oxidative stress and mitochondrial damage, which subsequently induce abnormal glycolysis and histone lactylation, making the chromatin structure of the PDE4B gene promoter region more open, thereby facilitating the binding of transcription factors and the formation of the transcription initiation complex, thus enhancing the transcription and expression of PDE4B.

Angiotensinogen (AGT), the initiating molecule of the Renin‐Angiotensin System (RAS), plays a crucial role in blood pressure regulation and maintenance of cardiovascular function. Through a series of enzymatic reactions, this system converts AGT into Angiotensin II (Ang II), which exerts potent vasoconstrictive and cell proliferative effects, thereby participating in the pathological processes of various cardiovascular diseases such as hypertension, atherosclerosis, and heart failure [36, 37, 38]. Our research found that under the pathological conditions of OSA combined with hypertension, PDE4B positively regulates AGT expression, and knocking out AGT improves the reprogramming of PASMCs. FUS is a multifunctional RNA‐binding protein involved in multiple processes such as transcriptional regulation, mRNA processing, and transportation. Its nuclear import is crucial for the precise regulation of gene expression, and this process is typically regulated by various factors, including post‐translational modifications like phosphorylation and ubiquitination [39, 40]. Our research discovered that PDE4B affects the phosphorylation status of FUS, thereby influencing its subcellular localization. Moreover, FUS not only influences the reprogramming of PASMCs; its P1 site also binds to AGT, thereby promoting its transcription and translation.

In summary, in OSA, histone lactylation induced by oxidative stress in PASMCs promotes the expression of PDE4B, which subsequently drives the development and progression of hypertension through the PDE4B/FUS/AGT signaling pathway. Upon knockout of PDE4B, CIH‐induced phosphorylation and nuclear localization of FUS are diminished, thereby suppressing AGT expression and mitigating PASMC reprogramming as well as pulmonary artery remodeling and right ventricular hypertrophy. Therefore, targeting PDE4B may represent an effective novel strategy for the treatment of OSA‐associated hypertension.

## Author Contributions

Li Yang, Qing Ni, Yan He and Shijie Liu contributed to the conception; Lulu Gan, Anni Dai, Yang Hu, Qian Liu, and Xueling Yang contributed to the design; Jiqian Li, Yi Tao, Yunyu Li, and Mingyue Xu analyzed and interpreted the data; Li Yang and Qing Ni drafted the article; Li Yang critically revised it for important intellectual content. All authors approved the final version for publication.

## Ethics Statement

This study has been approved by the Ethics Committee of The Affiliated Yan’an Hospital of Kunming Medical University Kunming Hypertension Center. All animal experiments were approved by The Affiliated Yan’an Hospital of Kunming Medical University Kunming Hypertension Center (approval no. 2022‐023).

## Consent

The authors have nothing to report.

## Conflicts of Interest

The authors declare no conflicts of interest.

## Supporting information


**Figure S1:** Knockout of PDE4B improves OSA combined with hypertension phenotype. (A) Representative images of rat lung tissue after 4 weeks of AAV1.PDE4B treatment, showing green fluorescence indicating AAV1.PDE4B expression. Scale bar: 100 μm. (B, C) Analysis of PDE4B mRNA (B) and protein (C) expression levels in lung homogenates from rats treated with AAV1.Luc or AAV1.PDE4B. Data are presented as mean ± SD (*n* = 6 per group). Statistical analysis was performed using an unpaired Student's *t*‐test. ****p < 0.0001. (D) Quantitative analysis of WGA‐stained cardiomyocytes assessing hypertrophy. Scale bar: 50 μm. Data are presented as mean ± SD (*n* = 6 per group). Statistical analysis was performed using an unpaired Student's *t*‐test. **p < 0.01, ****p < 0.0001.

**Figure S2:** EP300 regulates histone lactylation rather than histone acetylation. (A) Analysis of EP300 protein expression and quantification in PASMCs treated with different concentrations of 2‐DG or oxamate. Data are presented as mean ± SD (*n* = 3 per group). Statistical analysis was performed using one‐way ANOVA followed by Tukey's post hoc test. Ns = no significance. (B, C) Analysis of EP300 mRNA (B) and protein (C) expression in PASMCs treated with si‐PDE4B or not. Data are presented as mean ± SD (*n* = 3 per group). Statistical analysis was performed using an unpaired Student's *t*‐test. **p < 0.01. (D) ChIP‐qPCR analysis of H3K27ac status at the PDE4B genomic region in PASMCs. Data are presented as mean ± SD (*n* = 3 per group). Statistical analysis was performed using an unpaired Student's *t*‐test. (E) RT‐PCR analysis of PDE4B mRNA stability in PASMCs, with Actinomycin D used to block transcription. Data are presented as mean ± SD (*n* = 3 per group). Statistical analysis was performed using two‐way ANOVA followed by Bonferroni post hoc test.

**Figure S3:** Bioinformatic predictions. (A) Schematic diagram of interacting proteins of PDE4B predicted by the BioGRID database. (B) Schematic diagram of RNA‐binding proteins (RBPs) interacting with AGT predicted by the ENCORI database. (C) Schematic diagram of phosphorylation sites in FUS predicted by NetPhos 3.1.

**Figure S4:** Bioinformatic predictions. (A) Analysis of the human and rat bidirectional promoter of AGT revealing several FUS‐responsive elements (underlined in red). (B) Prediction of FUS binding sites using the ATtRACT website.

**Figure S5:** AGT/Ang II expression localization. (A) AGT/Ang II expression localization by IF staining in the lung tissue samples. Data are presented as mean ± SD (*n* = 3 per group). (B) AGT/Ang II expression localization by WB in the lung tissue samples. Data are presented as mean ± SD (*n* = 3 per group).

**Table S1:** Targeted sequences of siRNAs.
**Table S2:** qRT‐PCR primer sequences.

## Data Availability

The data that support the findings of this study are available from the corresponding author upon reasonable request.
