# Supplementary figures and images for: OSA Initiates Histone Lactylation That Drives PDE4B/FUS/AGT Axis to Pulmonary Hypertension
Source: Cell Prolif. 2025 Nov 17;59(5):e70145. doi: 10.1111/cpr.70145 (PMC13114777; doi:10.1111/cpr.70145)

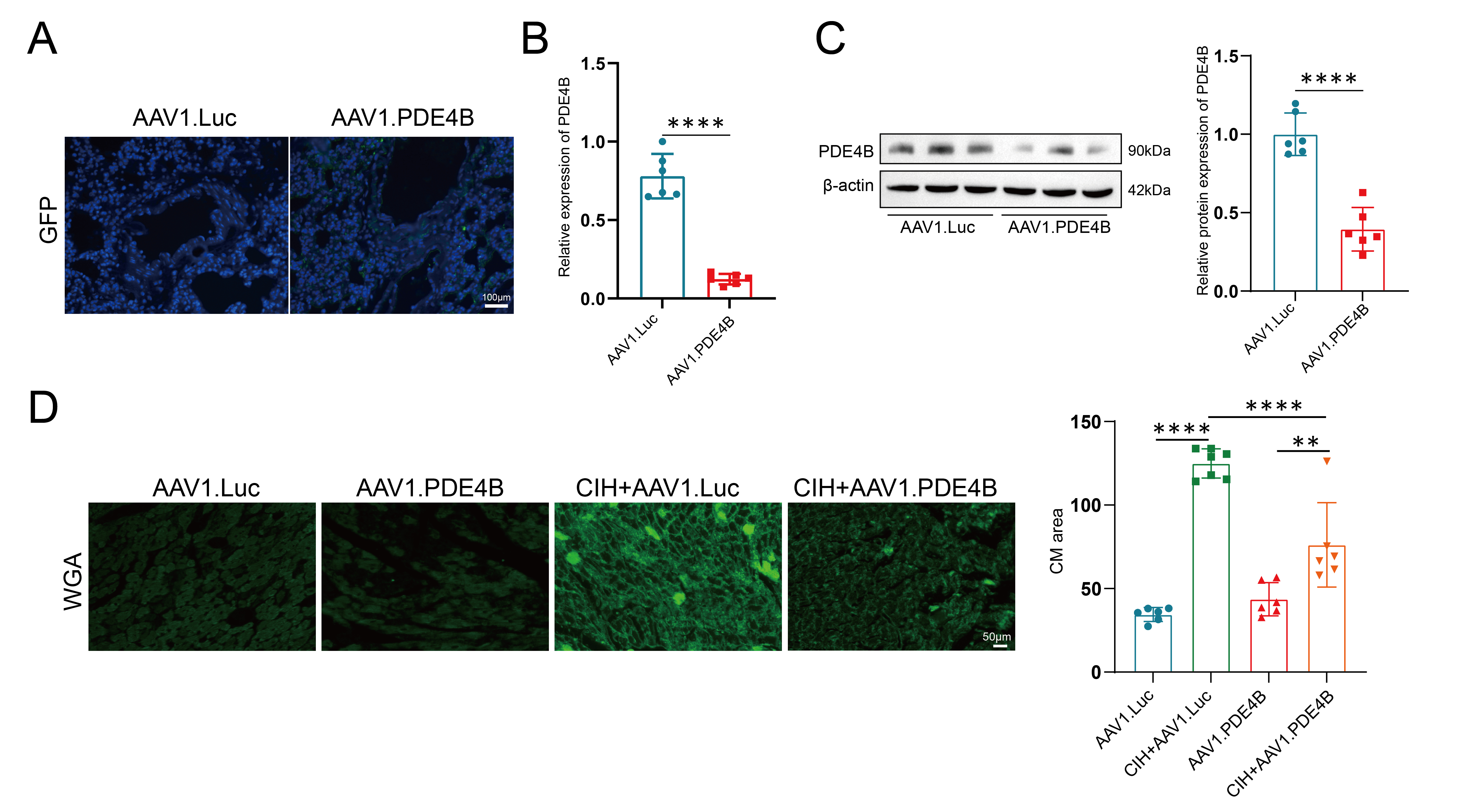

Supplement: Supplementary file 1 — Figure S1: Knockout of PDE4B improves OSA combined with hypertension phenotype. (A) Representative images of rat lung tissue after 4 weeks of AAV1.PDE4B treatment, showing green fluorescence indicating AAV1.PDE4B expression. Scale bar: 100 μm. (B, C) Analysis of PDE4B mRNA (B) and protein (C) expression levels in lung homogenates from rats treated with AAV1.Luc or AAV1.PDE4B. Data are presented as mean ± SD (n = 6 per group). Statistical analysis was performed using an unpaired Student's t‐test. ****p < 0.0001. (D) Quantitative analysis of WGA‐stained cardiomyocytes assessing hypertrophy. Scale bar: 50 μm. Data are presented as mean ± SD (n = 6 per group). Statistical analysis was performed using an unpaired Student's t‐test. **p < 0.01, ****p < 0.0001. [file CPR-59-e70145-s003.jpg]

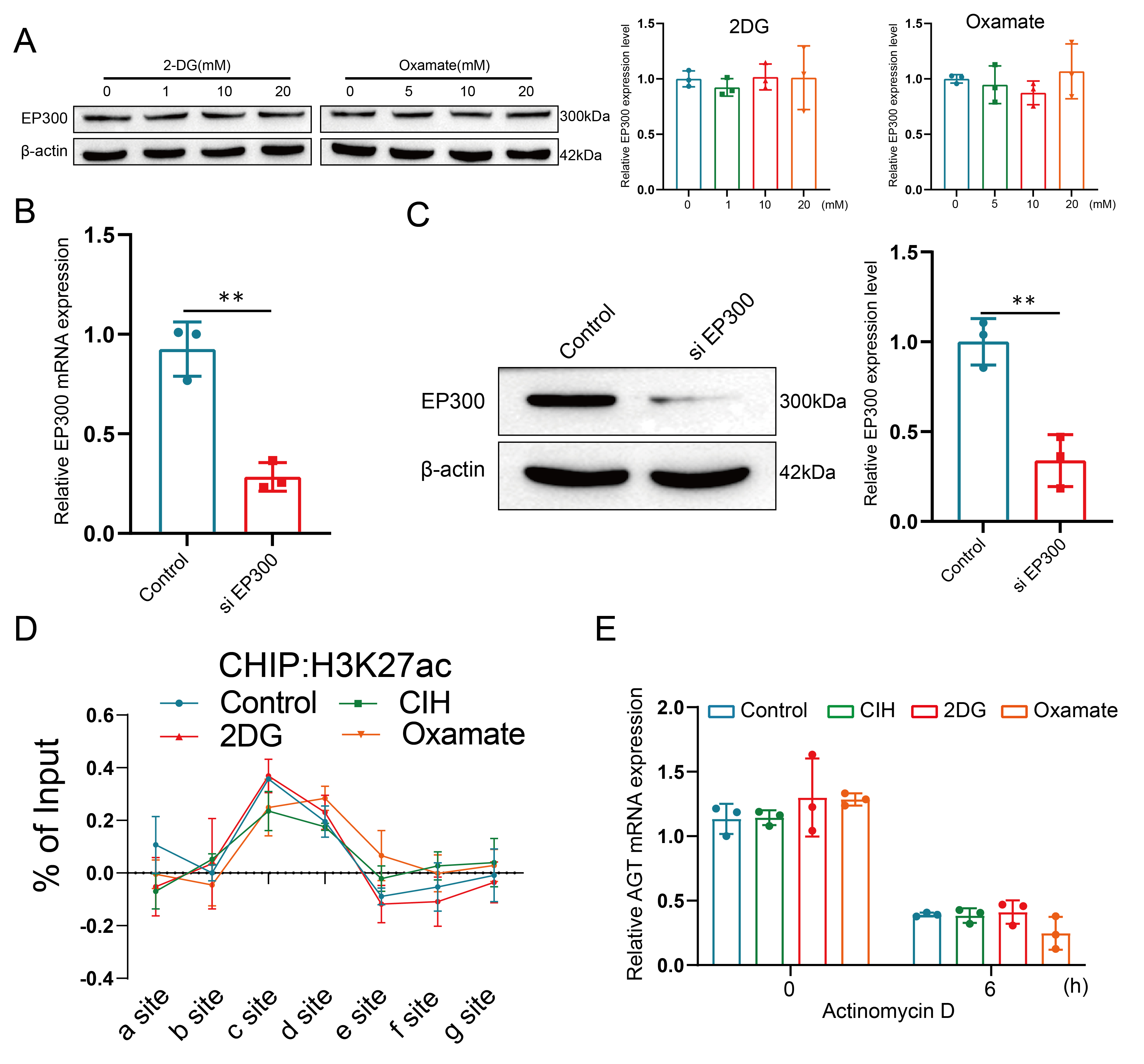

Supplement: Supplementary file 2 — Figure S2: EP300 regulates histone lactylation rather than histone acetylation. (A) Analysis of EP300 protein expression and quantification in PASMCs treated with different concentrations of 2‐DG or oxamate. Data are presented as mean ± SD (n = 3 per group). Statistical analysis was performed using one‐way ANOVA followed by Tukey's post hoc test. Ns = no significance. (B, C) Analysis of EP300 mRNA (B) and protein (C) expression in PASMCs treated with si‐PDE4B or not. Data are presented as mean ± SD (n = 3 per group). Statistical analysis was performed using an unpaired Student's t‐test. **p < 0.01. (D) ChIP‐qPCR analysis of H3K27ac status at the PDE4B genomic region in PASMCs. Data are presented as mean ± SD (n = 3 per group). Statistical analysis was performed using an unpaired Student's t‐test. (E) RT‐PCR analysis of PDE4B mRNA stability in PASMCs, with Actinomycin D used to block transcription. Data are presented as mean ± SD (n = 3 per group). Statistical analysis was performed using two‐way ANOVA followed by Bonferroni post hoc test. [file CPR-59-e70145-s004.jpg]

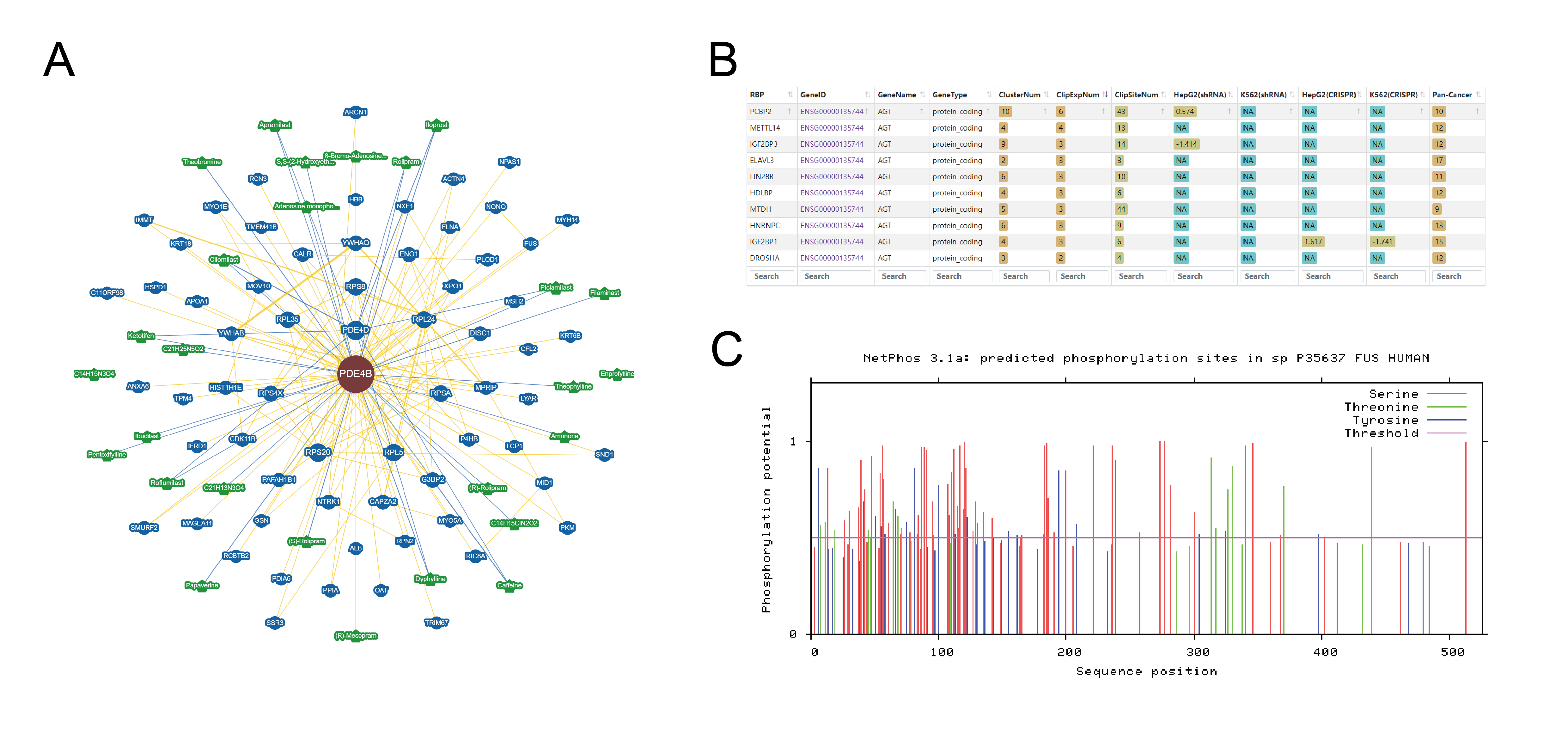

Supplement: Supplementary file 3 — Figure S3: Bioinformatic predictions. (A) Schematic diagram of interacting proteins of PDE4B predicted by the BioGRID database. (B) Schematic diagram of RNA‐binding proteins (RBPs) interacting with AGT predicted by the ENCORI database. (C) Schematic diagram of phosphorylation sites in FUS predicted by NetPhos 3.1. [file CPR-59-e70145-s001.jpg]

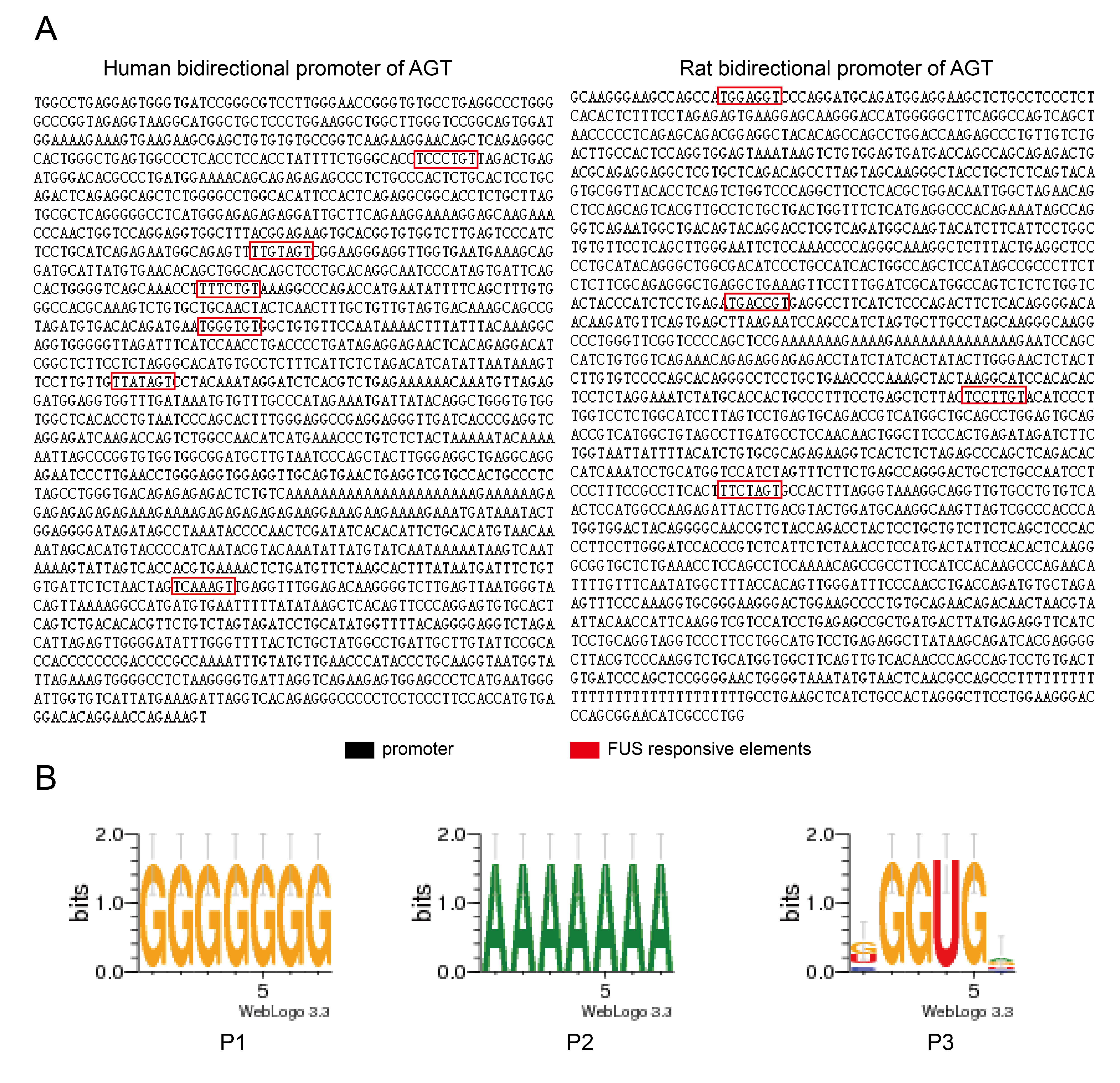

Supplement: Supplementary file 4 — Figure S4: Bioinformatic predictions. (A) Analysis of the human and rat bidirectional promoter of AGT revealing several FUS‐responsive elements (underlined in red). (B) Prediction of FUS binding sites using the ATtRACT website. [file CPR-59-e70145-s005.jpg]

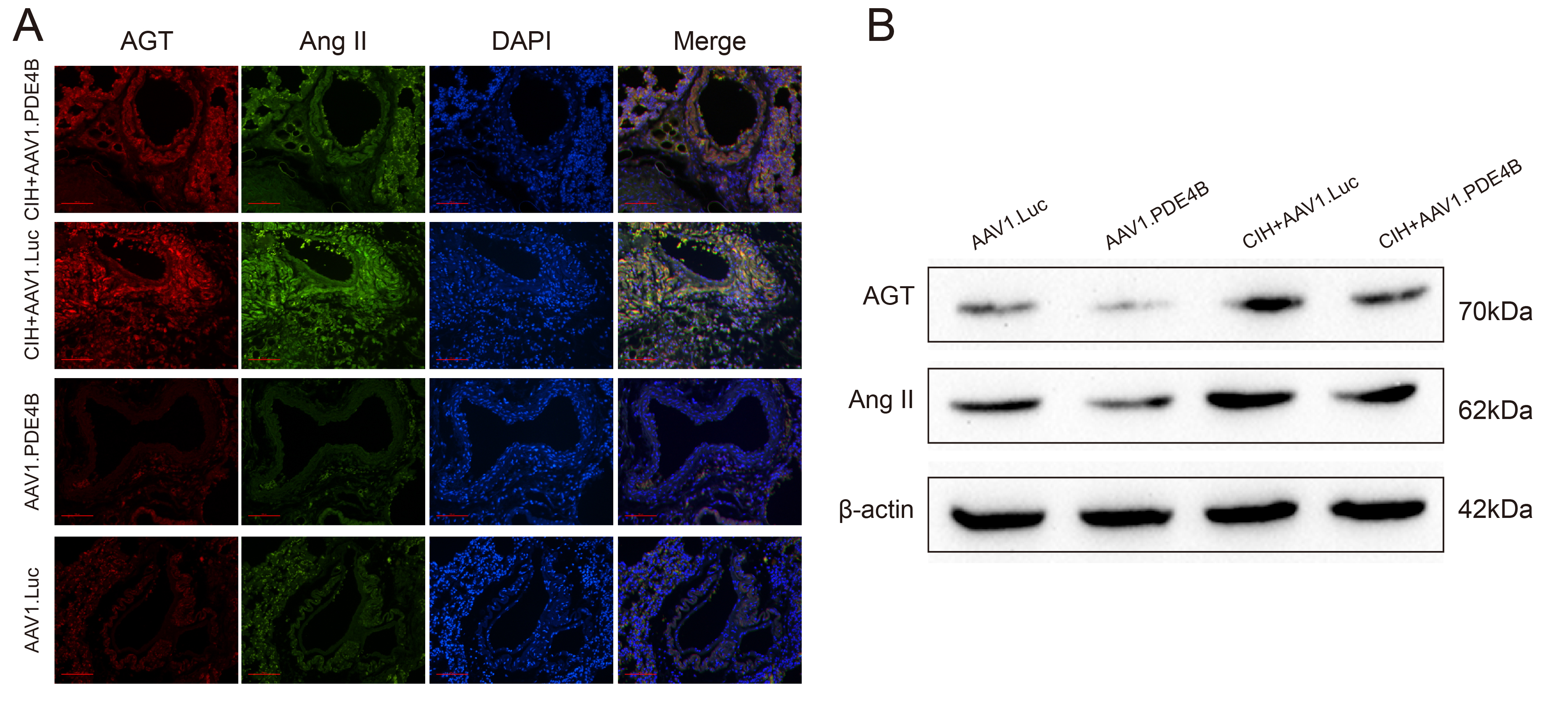

Supplement: Supplementary file 5 — Figure S5: AGT/Ang II expression localization. (A) AGT/Ang II expression localization by IF staining in the lung tissue samples. Data are presented as mean ± SD (n = 3 per group). (B) AGT/Ang II expression localization by WB in the lung tissue samples. Data are presented as mean ± SD (n = 3 per group). [file CPR-59-e70145-s002.jpg]
