# Supplementary material for: OSA Initiates Histone Lactylation That Drives PDE4B/FUS/AGT Axis to Pulmonary Hypertension
Source: Cell Prolif. 2025 Nov 17;59(5):e70145. doi: 10.1111/cpr.70145 (PMC13114777; doi:10.1111/cpr.70145)
Supplement: Supplementary file 6 — Table S1: Targeted sequences of siRNAs. Table S2: qRT‐PCR primer sequences. [file CPR-59-e70145-s006.docx]

**Table S1. Targeted sequences of siRNAs**

| **Organism** |  | **Sequence** |
| --- | --- | --- |
| **Human** | **si-PDE4B** | **5′-GACCTTCTAAAGACGTTTAAA-3′** |
|  | **si-AGT** | **5’-GGCCACCATCTTCTGCATC-3’** |
|  | **si-NC** | **5′UUCUCCGAACGUGUCACGUTT-3′** |

**Table S2. qRT-PCR primer sequences .**

| Organism | Gene | Forward primer (5'-3') | Reverse primer (5'-3') |
| --- | --- | --- | --- |
| Rat | GAPDH | GGTTGTCTCCTGCGACTTCA | GGTGGTCCAGGGTTTCTTACTC |
|  | PDE4B | TGCGTGTAATCCTCCAGCCT | TGACTGCAGACTAGACCTGGC |
|  | Col-1 | TGTTGGTCCTGCTGGCAAGAATG | GTCACCTTGTTCGCCTGTCTCAC |
|  | Fn | AGGCACAAGGTCCGAGAAGAGG | GGTCAAAGCATGAGTCATCCGTAGG |
| Human | GAPDH | GCTCTCTGCTCCTCCTGTTC | GACTCCGACCTTCACCTTCC |
|  | PDE4B | ACGGTGGCTCATACATGCT | GTACCAGTCCCGACGAAGAG |
|  | AGT | CAACACCTACGTCCACTTCCAA | TGTTGTCCACCCAGAACTCCT |
|  | EP300 | AGCCAAGCGGCCTAAACTC | TCACCACCATTGGTTAGTCCC |
